# Multi-Omics Analysis Identifies Gut-Associated Metabolic Signatures Associated with Metformin-Mediated Protection Against LPS-Induced Lung Injury

**DOI:** 10.3390/ph19101643

**Published:** 2026-10-18

**Authors:** Meihui Cheng, Mi Zhang, Wei Wang, Xianxian Jia, Bin Cong

**Affiliations:** 1 Postdoctoral Research Station in Basic Medicine, Hebei Medical University, Shijiazhuang 050017, China; 2 Research Unit of Digestive Tract Microecosystem Pharmacology and Toxicology, Chinese Academy of Medical Sciences, Hebei Medical University, Shijiazhuang 050017, China; 3 Hebei Key Laboratory of Forensic Medicine, Collaborative Innovation Center of Forensic Medical Molecular Identification, College of Forensic Medicine, Hebei Medical University, Shijiazhuang 050017, China; 4 Department of Pathogen Biology, Institute of Basic Medicine, Hebei Medical University, Shijiazhuang 050017, China

**Keywords:** metformin, LPS-induced lung injury, gut-lung axis, metabolomics, gut microbiota

## Abstract

**Background/Objectives**: Lipopolysaccharide (LPS)-induced lung injury involves pulmonary inflammation, metabolic disturbance, and intestinal dysregulation. This study investigated whether metformin attenuates LPS-induced lung and colonic injury and characterized associated gut microbiota–metabolite changes. **Methods**: Male C57BL/6J mice were assigned to control (CON), LPS, metformin 200 mg/kg (MET 200 mg/kg), and metformin 400 mg/kg (MET 400 mg/kg) groups. Metformin was administered by gavage for 7 days before intranasal LPS challenge. Lung and colon injury were assessed by histology, immunostaining, ELISA, and RT-qPCR. Untargeted serum metabolomics, colonic-content metabolomics, and colonic-content shotgun metagenomics were performed in the CON, LPS, and MET 400 mg/kg groups. Differential-feature, pathway-enrichment, and exploratory Spearman correlation analyses were conducted. **Results**: Metformin reduced LPS-induced lung injury, inflammatory cytokine production, F4/80- and MPO-positive immune-cell infiltration, and colonic inflammatory and barrier-related abnormalities. Serum metabolomics identified 468 differential features in LPS versus CON and 286 in MET versus LPS, with 218 overlapping features. Colonic-content metabolomics identified 1000 and 554 differential features, respectively, with 328 overlapping features. Eight metabolites showed metformin-associated directional shifts toward control levels in both serum and colonic contents. Metagenomics showed metformin-associated remodeling of gut microbial composition and predicted gene-content functional potential. Exploratory integrated analyses linked metformin-responsive genera and shared metabolites with pulmonary inflammatory indicators. **Conclusions**: Metformin was associated with attenuated LPS-induced lung and colonic injury and coordinated gut-associated microbiota–metabolite remodeling. Because the study did not include a metformin-only group or causal perturbation experiments, these findings identify candidate associations rather than demonstrating a causal gut microbiota–metabolite–lung mechanism.

## 1. Introduction

Acute lung injury (ALI) and acute respiratory distress syndrome (ARDS) are severe inflammatory disorders characterized by rapid respiratory dysfunction, inflammatory cytokine release, and immune-cell infiltration [1,2]. Lipopolysaccharide (LPS), a major component of Gram-negative bacterial outer membranes, is widely used to establish experimental ALI models because it activates innate immune responses and reproduces key features of infection-associated lung injury [3,4]. Although the lung is the principal target organ, ALI is increasingly viewed as a systemic inflammatory disorder. Recent gut–lung axis studies indicate that microbial dysbiosis and microbial metabolites can influence pulmonary immune responses [5,6,7,8]. Therefore, analysis of intestinal microbiota and metabolism may provide a useful perspective for understanding LPS-induced lung injury.

Metformin is a first-line antidiabetic drug with biological activities that extend beyond glucose lowering [9,10,11,12,13]. In addition to regulating host energy metabolism, metformin has been reported to exert anti-inflammatory effects and to modulate gut microbial composition and function [11,12,14,15,16,17,18]. A recent study further demonstrated that metformin could ameliorate LPS-induced and SARS-CoV-2-induced ARDS, supporting its protective potential in pulmonary inflammatory injury [19]. This evidence indicates that direct host-intrinsic mechanisms, including effects on mitochondrial metabolism and NLRP3 inflammasome activation, can contribute to the pulmonary protection produced by metformin [19]. In addition, direct host-intrinsic mechanisms are supported by prior experimental studies; metformin attenuated LPS-induced epithelial senescence by activating autophagy [20] and alleviated endotoxemia-induced acute lung injury through AMPK-dependent suppression of mTOR [21]. Because orally administered metformin accumulates at relatively high concentrations in the gastrointestinal tract, the intestine is considered an important site of its pharmacological action [14,22]. These properties suggest that metformin may protect against inflammatory injury partly through intestinal microbial and metabolic regulation. However, whether metformin attenuates LPS-induced pulmonary inflammation through coordinated remodeling of the gut microbiota and host-microbe metabolites remains insufficiently understood.

Metabolites are important mediators of communication between intestinal microbes and the host [23,24,25,26,27]. Microbial and host-microbe co-metabolites, including indole derivatives, phenolic acids, bile acid-related metabolites, fatty acid-related metabolites, and amino acid metabolites, can enter the circulation and regulate epithelial barrier integrity, immune-cell activation, and cytokine production at distal sites [28,29,30,31,32]. Serum metabolites may reflect systemic inflammatory status, whereas colonic-content metabolites directly reflect the intestinal luminal environment. Therefore, from a gut–lung axis perspective, integrating microbiota and metabolomics through combined analysis of serum metabolomics, colonic-content metabolomics, and gut metagenomics may help identify microbial and metabolic signals associated with metformin-mediated protection [6,7,8].

In this study, we established an LPS-induced inflammatory injury model to evaluate the protective effects of metformin from a gut-lung axis perspective. Mice were assigned to control (CON), LPS, MET (200 mg/kg), and MET (400 mg/kg) groups for pathological and inflammatory assessment of lung and colon tissues. To explore the intestinal microbial and metabolic responses associated with metformin intervention, untargeted serum metabolomics, colonic-content metabolomics, and colonic-content metagenomics were performed in CON, LPS, and MET (400 mg/kg) mice. By integrating pathological indicators, microbial taxa, predicted microbial functional pathways, and shared gut–serum metabolites, we aimed to characterize the multi-system response associated with metformin-mediated attenuation of LPS-induced lung inflammation, while recognizing that this design identifies associations rather than proving a causal gut microbiota–metabolite–lung pathway.

## 2. Results

### 2.1. Metformin Attenuated LPS-Induced Lung Inflammation and Colonic Injury

**Figure 1:**
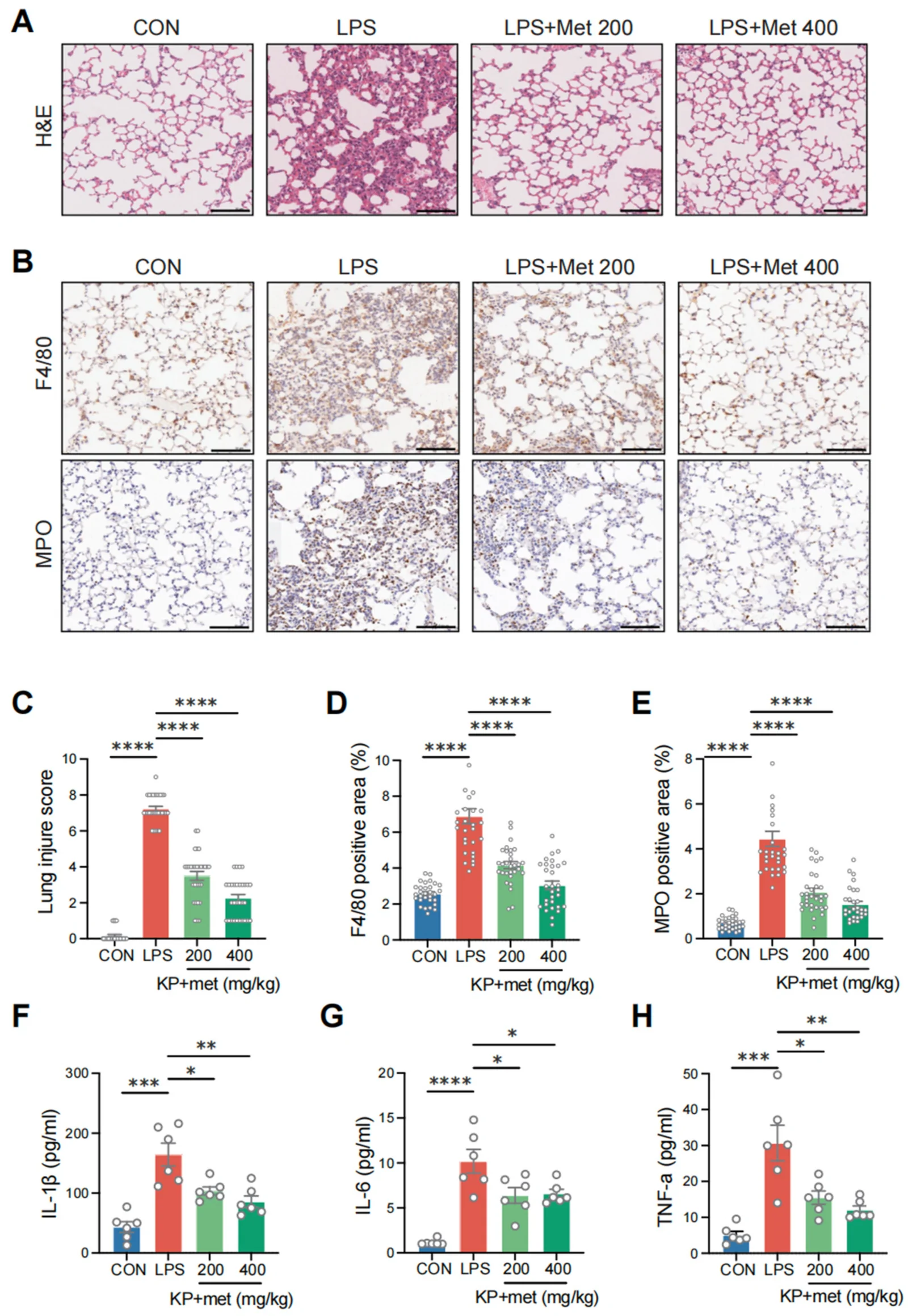
Metformin attenuated LPS-induced pulmonary inflammation and tissue injury. (**A**) Representative H&E-stained lung sections from the CON, LPS, MET (200 mg/kg), and MET (400 mg/kg) groups. (**B**) Representative immunohistochemical staining for F4/80 and MPO in lung tissues. (**C**) Quantification of lung histopathological scores. Representative images are shown in panels (**A**,**B**). Quantitative analyses were conducted in six biological replicate mice per group, with five randomly selected fields blindly analyzed per mouse. (**D**,**E**) Quantification of the F4/80- and MPO-positive staining areas. (**F**–**H**) ELISA-based determination of IL-1β, IL-6, and TNF-α levels in lung tissue homogenates. In panels (**C**–**E**), each white dot represents one randomly selected field (five fields per mouse, six mice per group, n = 30 fields per group). In panels (**F**–**H**), each white dot represents one individual mouse (n = 6 per group). Data are shown as mean ± SEM. * *p* < 0.05, ** *p* < 0.01, *** *p* < 0.001, **** *p* < 0.0001; ns, not significant, compared with the LPS group. Scale bars for (**A**,**B**): 100 μm.

To evaluate whether metformin protected against LPS-induced inflammatory injury, mice were assigned to four groups: CON, LPS, MET (200 mg/kg), and MET (400 mg/kg). Compared with CON mice, LPS challenge markedly aggravated lung injury, as shown by increased H&E pathological scores, elevated lung homogenate IL-1β, IL-6, and TNF-α levels, and increased F4/80- and MPO-positive staining areas (Figure 1A–H). Both metformin doses reduced these LPS-induced inflammatory changes. MET (200 mg/kg) significantly decreased the H&E score, IL-1β, IL-6, TNF-α, F4/80-positive area, and MPO-positive area, while MET (400 mg/kg) produced a stronger or comparable reduction across most lung injury indicators. These data showed that metformin effectively alleviated LPS-induced pulmonary inflammation.

**Figure 2:**
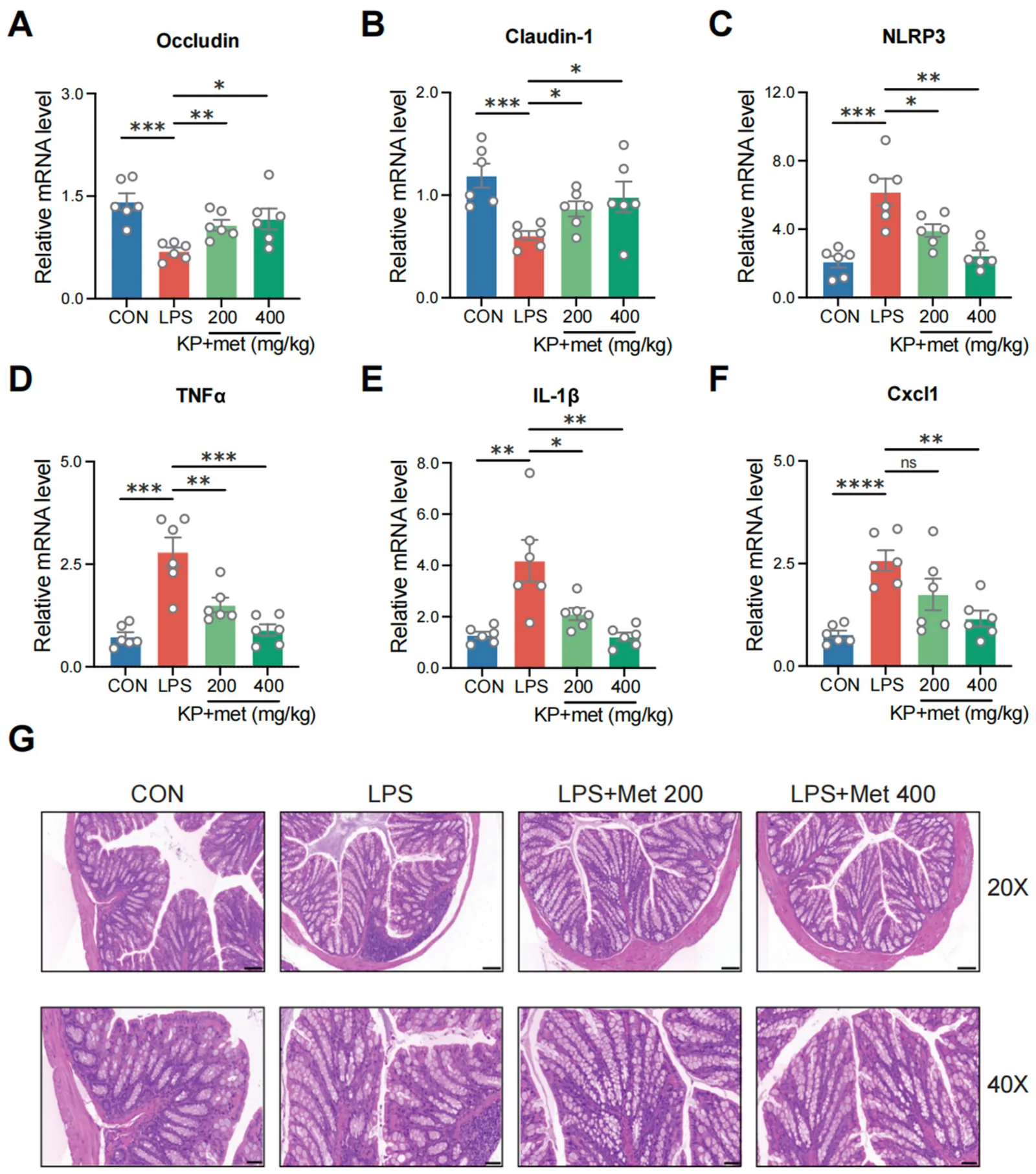
Metformin improved LPS-induced colonic barrier impairment and inflammation. (**A**–**F**) Relative mRNA expression levels of *Occludin*, *Claudin*, *Nlrp3*, *Tnfa*, *Il1b*, and *Cxcl1* in colon tissues were determined by RT-qPCR. (**G**) Representative H&E-stained colon sections from the CON, LPS, MET (200 mg/kg), and MET (400 mg/kg) groups. Images are representative of six biological replicate mice per group. Each white dot represents one individual mouse (n = 6 per group). Data are shown as mean ± SEM. * *p* < 0.05, ** *p* < 0.01, *** *p* < 0.001, **** *p* < 0.0001; ns, not significant, compared with the LPS group.

Consistent with the lung phenotype, LPS also induced colonic inflammatory and barrier-related abnormalities. LPS increased the expression of inflammatory genes, including *Tnfa*, *Il1b*, *Cxcl1*, and *Nlrp3*, and reduced epithelial barrier markers, including *Occludin* and *Claudin* (Figure 2A–F). Metformin treatment reversed these alterations to different extents. MET (400 mg/kg) significantly reduced *Tnfa*, *Il1b*, *Cxcl1*, and *Nlrp3* expression and restored *Occludin* and *Claudin* levels, whereas MET (200 mg/kg) significantly improved most inflammatory and barrier markers, with a weaker effect on *Cxcl1*. These findings were further supported by H&E staining of colonic tissues (Figure 2G). Collectively, these results indicate that metformin not only protects against LPS-induced pulmonary inflammation but also alleviates intestinal inflammatory damage and barrier disruption.

### 2.2. Serum Metabolomics Revealed Metformin-Associated Shifts in LPS-Disrupted Metabolic Profiles

**Figure 3:**
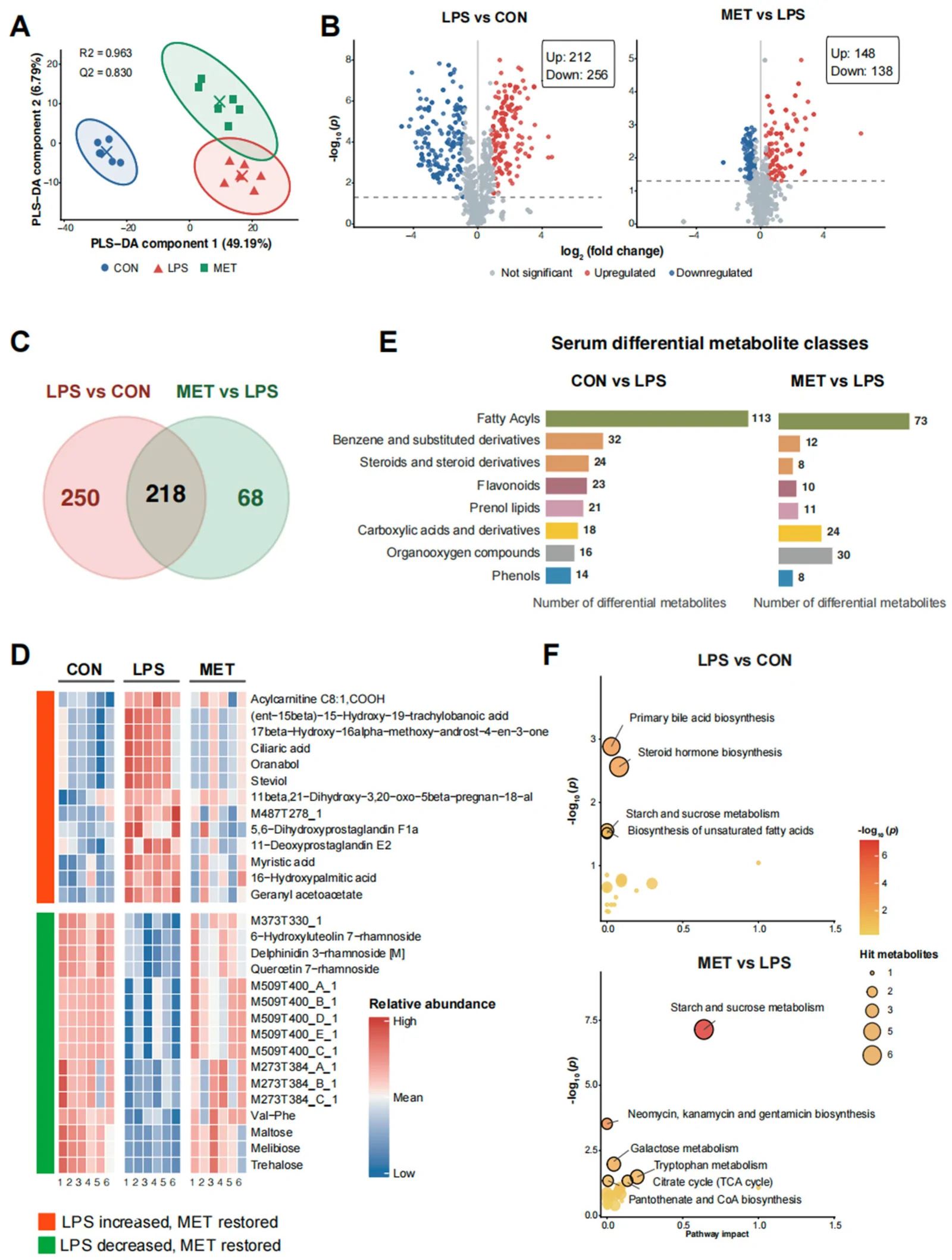
Serum metabolomic profiling revealed metformin-associated shifts in LPS-induced metabolic disturbance. (**A**) PLS-DA score plot of serum metabolomic profiles from CON, LPS, and MET (400 mg/kg) groups. (**B**) Volcano plots showing differential serum metabolic features in LPS versus CON and MET (400 mg/kg) versus LPS comparisons. Red and blue dots indicate upregulated and downregulated features. (**C**) Venn diagram showing overlapping differential serum features between the LPS versus CON and MET (400 mg/kg) versus LPS comparisons. (**D**) Heatmap of representative metformin-responsive serum metabolites. (**E**) Chemical classification of differential serum metabolites. Each colored bar represents a different metabolite class. (**F**) Summary diagram of the MetaboAnalyst 6.0-based pathway analysis of differential serum metabolites in LPS versus CON and MET (400 mg/kg) versus LPS comparisons. Differential metabolites were screened using VIP 1, nominal *p* < 0.05 and fold change >1.2 or <0.83.

Untargeted serum metabolomics was performed in CON, LPS, and MET (400 mg/kg) mice to determine whether systemic metabolic changes were associated with the protective effect of metformin. PLS-DA score plots showed separation among the three groups, indicating that LPS challenge and metformin intervention produced distinct serum metabolic profiles (Figure 3A). Volcano plot analysis identified 468 differential serum features in LPS versus CON, including 212 upregulated and 256 downregulated features. In MET versus LPS, 286 differential features were detected, including 148 upregulated and 138 downregulated features (Figure 3B). Venn analysis showed that 218 serum differential features overlapped between the two comparisons (Figure 3C and Supplementary Table S1). Heatmap analysis further demonstrated that these serum metabolites displayed LPS-induced disturbance and metformin-associated shifts toward the CON profile (Figure 3D).

Classification analysis showed that the major differential serum metabolite classes included fatty acyls, benzene and substituted derivatives, steroids and steroid derivatives, flavonoids, and prenol lipids (Figure 3E). After importing the differential metabolites into MetaboAnalyst 6.0 for pathway enrichment analysis, the results revealed that LPS predominantly perturbed four metabolic pathways: primary bile acid biosynthesis, steroid hormone biosynthesis, biosynthesis of unsaturated fatty acids, and starch and sucrose metabolism. Metformin intervention was associated with changes in starch and sucrose metabolism, steroid-related metabolism, galactose metabolism, tryptophan metabolism, pantothenate and CoA biosynthesis, and the tricarboxylic acid cycle (Figure 3F). Together, the serum metabolomics results indicated that metformin treatment was associated with broad systemic metabolic remodeling after LPS challenge.

### 2.3. Colonic-Content Metabolomics Identified Extensive Metabolic Remodeling Associated with Metformin

**Figure 4:**
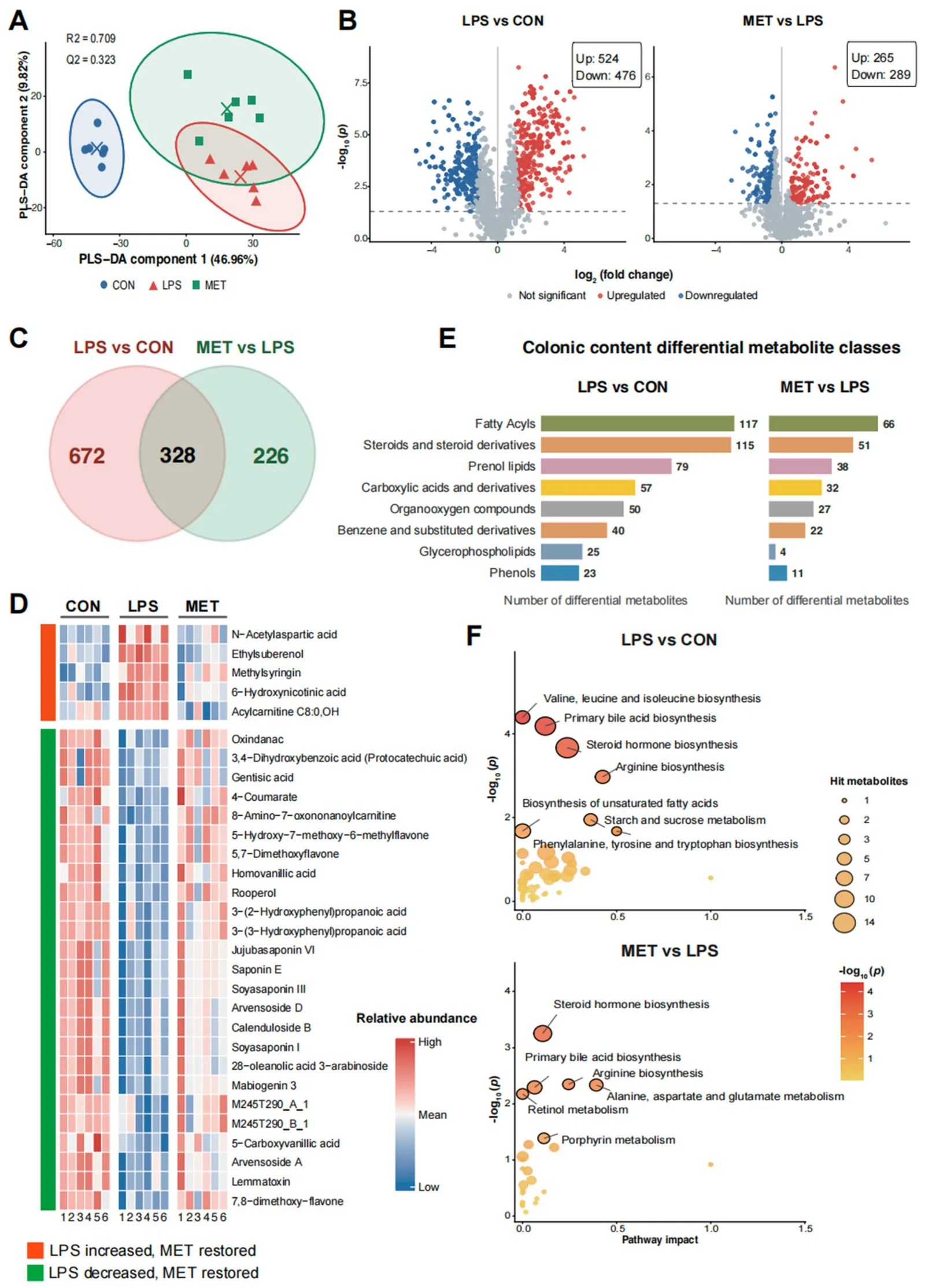
Colonic-content metabolomics identified intestinal metabolic remodeling after LPS challenge and metformin treatment. (**A**) PLS-DA score plot of colonic-content metabolomic profiles from CON, LPS and MET (400 mg/kg) groups. (**B**) Volcano plots showing differential colonic-content metabolic features in LPS versus CON and MET (400 mg/kg) versus LPS comparisons. (**C**) Venn diagram showing overlapping differential colonic-content features between the LPS versus CON and MET (400 mg/kg) versus LPS comparisons. (**D**) Heatmap of representative metformin-responsive colonic-content metabolites. (**E**) Chemical classification of differential colonic-content metabolites. Each colored bar represents a different metabolite class. (**F**) Summary diagram of the MetaboAnalyst 6.0-based pathway analysis of differential colonic-content metabolites.

To determine whether intestinal luminal metabolites were also altered, untargeted metabolomics was performed using colonic contents from CON, LPS, and MET (400 mg/kg) mice. PLS-DA analysis showed metabolic separation among the three groups (Figure 4A). LPS versus CON yielded 1000 differential metabolic features, including 524 upregulated and 476 downregulated features. MET versus LPS yielded 554 differential features, including 265 upregulated and 289 downregulated features (Figure 4B). Of these, 328 features overlapped between the two comparisons (Figure 4C and Supplementary Table S2). Using a directional-response criterion, 112 colonic-content metabolites were identified as LPS-altered and oppositely regulated by metformin relative to LPS (Supplementary Figure S1). A heatmap of the top 30 representative metabolites showed that LPS caused a distinct intestinal metabolic pattern, whereas MET shifted these metabolites toward the CON profile (Figure 4D).

Chemical classification indicated that the major differential metabolite classes included fatty acyls, steroids and steroid derivatives, prenol lipids, carboxylic acids and derivatives, and organooxygen compounds (Figure 4E). Differential metabolites were imported into MetaboAnalyst 6.0 for pathway analysis. The results showed that LPS mainly affected valine, leucine and isoleucine biosynthesis, primary bile acid biosynthesis, steroid hormone biosynthesis, and arginine biosynthesis, whereas metformin treatment was associated with steroid hormone biosynthesis, arginine biosynthesis, alanine, aspartate and glutamate metabolism, primary bile acid biosynthesis, retinol metabolism, and porphyrin metabolism (Figure 4F). These results showed that metformin substantially remodeled the intestinal metabolic environment after LPS exposure.

### 2.4. Eight Metabolites Showed Synchronized Metformin-Associated Shifts in Serum and Colonic Contents

**Figure 5:**
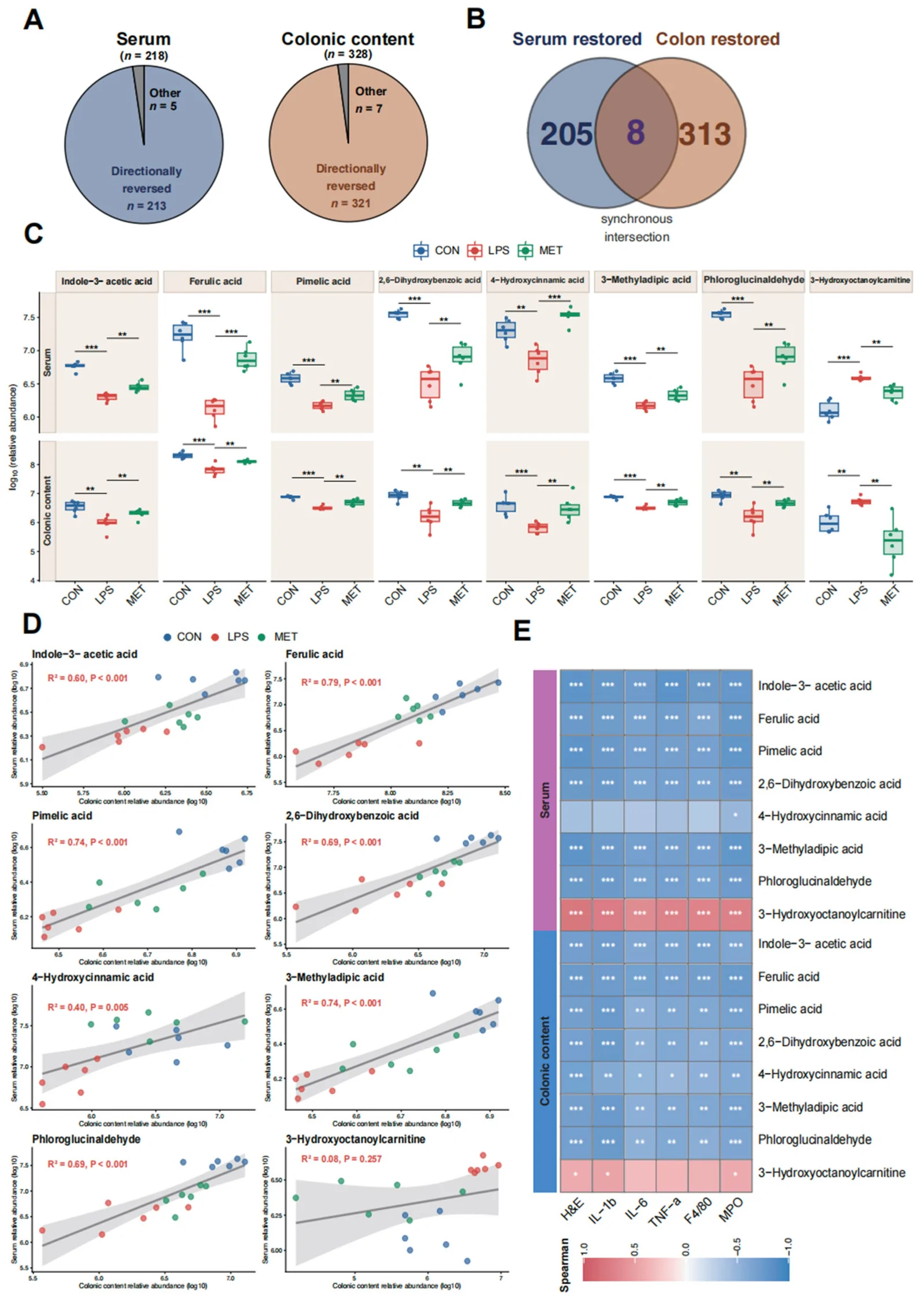
Identification of metabolites showing synchronized metformin-associated shifts in serum and colonic contents. (**A**,**B**) Integrated screening of directionally responsive metabolites in serum and colonic contents. The blue bars represent serum, and the orange bars represent colonic contents. (**C**) Quantification of the eight synchronized metabolites in serum and colonic contents across CON, LPS and MET (400 mg/kg) groups. (**D**) Correlation analysis between serum and colonic-content abundances of the shared metabolites. (**E**) Spearman correlation heatmap between shared metabolites and lung injury indicators, including H&E score, IL-1β, IL-6, TNF-α, F4/80 and MPO. * *p* < 0.05, ** *p* < 0.01, *** *p* < 0.001.

To identify metabolites potentially linking the intestinal and systemic metabolic responses, metformin-responsive metabolites from serum and colonic contents were compared (Figure 5A). Among 218 overlapping serum differential features, 213 were oppositely regulated by metformin relative to LPS. Among 328 overlapping colonic-content features, 321 showed the same directional pattern (Supplementary Tables S1 and S2). Intersection analysis identified eight metabolites that showed synchronized metformin-associated shifts in both serum and colonic contents: indole-3-acetic acid, ferulic acid, pimelic acid, 2,6-dihydroxybenzoic acid, 4-hydroxycinnamic acid, 3-methyladipic acid, phloroglucinaldehyde, and 3-hydroxyoctanoylcarnitine (Figure 5B).

Quantitative analysis showed that these eight metabolites changed significantly after LPS exposure and were oppositely regulated by MET in both serum and colonic contents (Figure 5C). Seven metabolites, including indole-3-acetic acid, ferulic acid, pimelic acid, 2,6-dihydroxybenzoic acid, 4-hydroxycinnamic acid, 3-methyladipic acid, and phloroglucinaldehyde, were decreased by LPS and increased by MET, whereas 3-hydroxyoctanoylcarnitine was increased by LPS and decreased by MET. Correlation analysis between paired colonic-content and serum abundance showed coordinated cross-compartment changes for these metabolites (Figure 5D). In addition, most of these metabolites were negatively correlated with lung inflammatory injury indicators, including H&E score, IL-1β, IL-6, TNF-α, F4/80, and MPO (Figure 5E). These results identified a set of gut-serum synchronized metabolites associated with metformin-mediated improvement of lung inflammation, without establishing their colonic origin or causal role.

### 2.5. Metformin Altered Gut Microbial Diversity, Composition and Functional Potential

**Figure 6:**
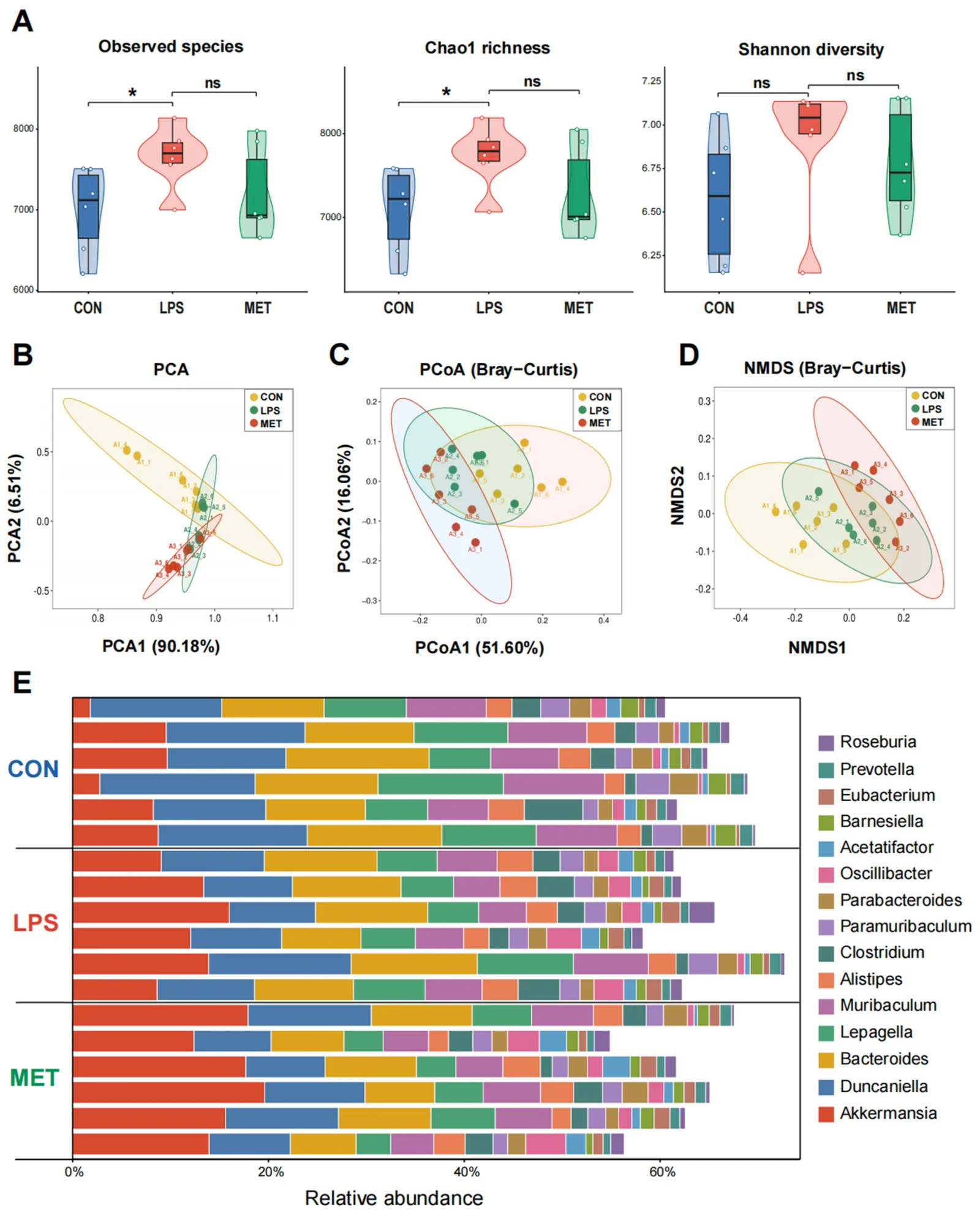
Metformin reshaped gut microbial diversity and genus-level composition in LPS-treated mice. (**A**) Alpha-diversity indices, including observed species, Chao1 richness, and Shannon diversity, in CON, LPS, and MET (400 mg/kg) groups. (**B**–**D**) Beta-diversity analysis of colonic-content metagenomes using PCA, Bray-Curtis PCoA, and Bray-Curtis NMDS. (**E**) Genus-level taxonomic composition of colonic-content microbiota across individual samples. Each point represents one mouse. * *p* < 0.05; ns, not significant.

Colonic-content metagenomic analysis was then used to evaluate whether the metabolic changes were accompanied by changes in the gut microbiota. Alpha-diversity analysis showed that LPS increased observed species and Chao1 richness compared with CON, whereas MET shifted these richness indices toward CON but did not reach statistical significance versus LPS after multiple-comparison adjustment (Figure 6A). Beta-diversity ordination showed separation among the three groups, indicating that LPS and MET (400 mg/kg) were associated with changes overall microbial community structure (Figure 6B–D). Genus-level composition analysis further showed that the dominant microbial taxa differed among CON, LPS, and MET (400 mg/kg) groups (Figure 6E).

**Figure 7:**
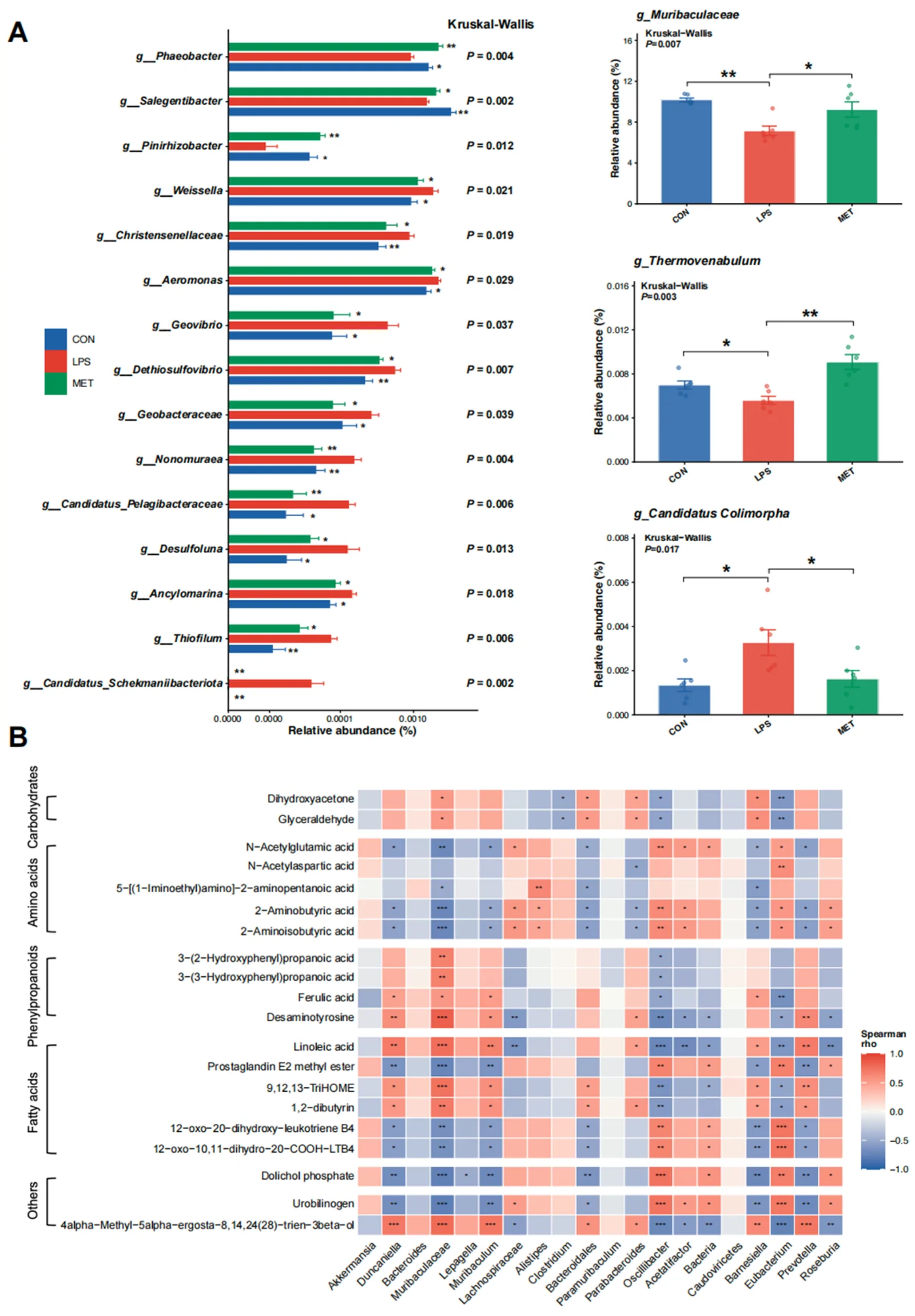
Metformin-regulated gut genera were associated with metformin-responsive colonic metabolites. (**A**) Relative abundance patterns of differential genera altered by LPS and directionally modulated by MET (400 mg/kg) at the nominal screening level. Bars show mean ± SEM. (**B**) Spearman correlation heatmap between the top 20 abundant genera and the top 20 MET-responsive endogenous colonic metabolites. Red indicates positive correlations and blue indicates negative correlations. * *p* < 0.05, ** *p* < 0.01, *** *p* < 0.001.

Differential genus analysis identified 18 genera with an LPS-altered and MET-reversed pattern at the nominal screening level (Figure 7A). The corresponding Benjamini-Hochberg-adjusted pairwise q values did not fall below 0.05; therefore, these taxa are presented as exploratory directional-response candidates. Among these, several genera were depleted by LPS and increased after MET treatment, including g_Muribaculaceae, g_Thermovenabulum, g_Phaeobacter, g_Salegentibacter, and g_Pinirhizobacter. Other genera were enriched by LPS and reduced after MET treatment, including g_Candidatus Colimorpha, g_Nonomuraea, g_Weissella, and related taxa. Correlation analysis further showed that these metformin-responsive genera were coordinately associated with the metabolite changes, supporting a linked microbiota–metabolite response to treatment (Figure 7B). In parallel, metagenomic functional profiling identified pathway-level differences in predicted gene-content potential after metformin intervention. Metformin was associated with pathways such as protein processing, C5-branched dibasic acid metabolism, valine, leucine and isoleucine biosynthesis, arginine biosynthesis and pantothenate and CoA biosynthesis, whereas LPS was linked to pathway labels including PI3K-Akt signaling, necroptosis, insulin resistance, the sulfur relay system, and teichoic acid biosynthesis (Supplementary Figure S2). Because some KEGG pathway labels, such as PI3K-Akt signaling, necroptosis and insulin resistance, are derived from homologous gene annotations and are not direct evidence of mammalian signaling activity in microbes, these results should be interpreted as predicted functional potential rather than pathway activity.

### 2.6. Integrated Multi-Omics Analysis Linked Gut Microbiota, Shared Metabolites and Lung Inflammation

**Figure 8:**
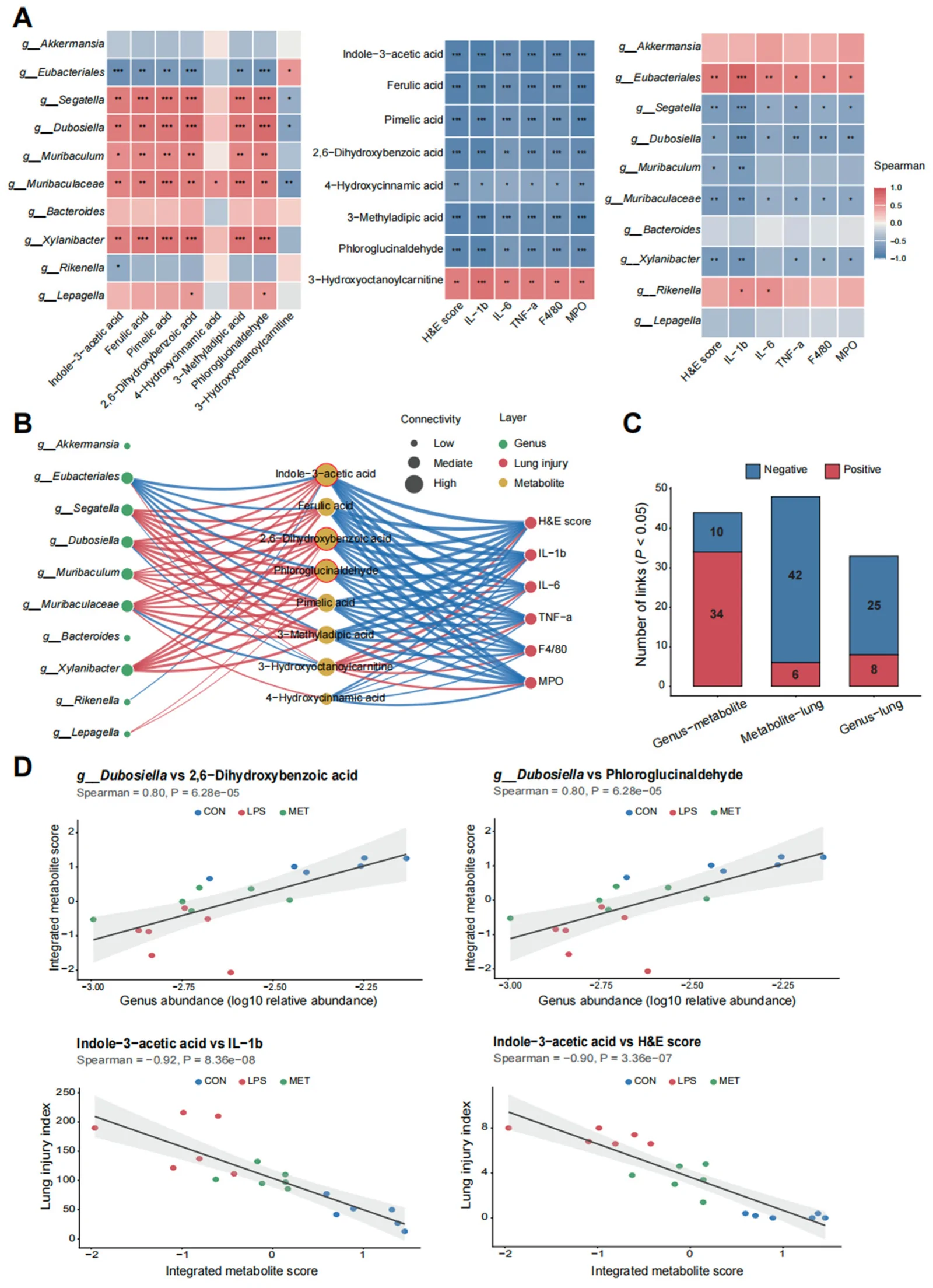
Integrated multi-omics analysis linked gut microbial genera, shared metabolites and lung inflammatory injury. (**A**) Spearman correlation heatmaps showing associations between top differential genera and eight common metformin-responsive metabolites, between common metformin-responsive metabolites and lung injury indices, and between differential genera and lung injury indices. (**B**) Multi-layer correlation network connecting differential gut genera, eight common metformin-responsive metabolites and lung injury indicators. Edges indicate nominally significant Spearman correlations (*p* < 0.05). Red edges indicate positive correlations, and blue edges indicate negative correlations. (**C**) Numbers of nominally significant positive and negative links among genus–metabolite, metabolite–lung, and genus–lung associations. (**D**) Representative scatter plots showing correlations between selected genera and metformin-responsive metabolites, and between metformin-responsive metabolites and lung injury indices. * *p* < 0.05, ** *p* < 0.01, *** *p* < 0.001.

Finally, we integrated the top differential genera, eight synchronized metformin-responsive metabolites, and six lung injury indicators to assess their cross-layer relationships. Correlation heatmaps showed that differential genera were associated with these metabolites, while the metabolites were associated with lung inflammatory injury indicators (Figure 8A). Network analysis further connected the three layers, consisting of gut genera, common metformin-responsive metabolites, and lung injury indices (Figure 8B). Most nominally significant genus-metabolite associations were positive, whereas most metabolite-lung injury associations were negative.

Quantitatively, 34 nominally significant positive and 10 nominally significant negative genus-metabolite correlations were detected, while 42 nominally significant negative and 6 nominally significant positive metabolite-lung injury correlations were observed (Figure 8C). The network identified indole-3-acetic acid, 2,6-dihydroxybenzoic acid, and phloroglucinaldehyde as high-degree hub metabolites, suggesting that these metabolites may mark coordinated relationships between gut microbial remodeling and pulmonary inflammatory status. Representative scatter plots illustrated inverse associations between several metformin-responsive metabolites and lung injury markers (Figure 8D). Overall, these multi-omics results support an association model in which metformin reshapes the gut microbiota and modulates metabolites both in the intestine and systemically, and these changes are associated with the attenuation of LPS-induced lung inflammation.

## 3. Discussion

In our study, metformin attenuated LPS-induced pulmonary inflammation and colonic injury, and these phenotypic improvements were accompanied by coordinated remodeling of serum metabolites, colonic-content metabolites, and gut microbial profiles. This finding is consistent with previous evidence that metformin can improve LPS-associated pulmonary inflammatory injury [19]. It also agrees with the gut–lung axis concept used in recent ALI studies, in which pulmonary inflammation is interpreted together with intestinal barrier damage, microbial dysbiosis, and metabolic disturbance [6,7,8]. Our data extend this framework by linking metformin treatment to a multi-layer intestinal response associated with reduced lung inflammatory indices.

The simultaneous improvement of lung and colon injury is important for interpreting the protective effect of metformin. LPS challenge increased pulmonary inflammatory indices and disrupted colonic inflammatory and barrier-related markers, indicating that lung inflammation occurred alongside intestinal mucosal disturbance. Metformin reversed these changes to different extents, with both doses improving pulmonary inflammation and the higher dose showing stronger or comparable effects for several tissue injury indicators. This pattern suggests that preservation of intestinal homeostasis may accompany, and possibly contribute to, the attenuation of pulmonary inflammation [6,7]. However, because the present study did not directly interrupt gut–lung signaling, intestinal protection should be interpreted as a metformin-associated response rather than definitive proof of causality.

The serum metabolomics results indicate that LPS induced a broad systemic metabolic disturbance that was partly normalized by metformin. The altered metabolite classes and enriched pathways involved fatty acyls, steroids and steroid derivatives, bile acid-related metabolism, unsaturated fatty acid biosynthesis, carbohydrate metabolism, tryptophan metabolism, pantothenate and CoA biosynthesis, and the tricarboxylic acid cycle. These pathways are closely connected with inflammatory signaling, energy metabolism, and immune-cell activation [30,33,34,35,36,37]. Therefore, the metformin-associated serum metabolic shift may reflect a transition from an inflammatory metabolic state toward a more regulated systemic profile. This interpretation is consistent with the known capacity of metformin to influence host metabolism and inflammatory responses [10,11,12,19].

Colonic-content metabolomics revealed extensive metabolic remodeling in the intestinal lumen. LPS altered a large number of colonic-content metabolic features, whereas metformin shifted many of them toward the control profile. Pathway analysis highlighted amino acid biosynthesis, primary bile acid biosynthesis, steroid hormone biosynthesis, arginine biosynthesis, retinol metabolism, and porphyrin metabolism. These changes suggest that the intestinal luminal environment is highly sensitive to LPS-induced inflammatory stress and metformin intervention. Because colonic-content metabolites can arise from diet, host secretion, microbial metabolism, and host-microbe co-metabolism, intestinal absorption, transit and inflammatory exudation, the observed profile should be interpreted as a composite luminal metabolic readout rather than direct evidence that specific microbes produced these metabolites [27].

A notable finding was the identification of eight metabolites showing synchronized metformin-associated shifts in both serum and colonic contents: indole-3-acetic acid, ferulic acid, pimelic acid, 2,6-dihydroxybenzoic acid, 4-hydroxycinnamic acid, 3-methyladipic acid, phloroglucinaldehyde, and 3-hydroxyoctanoylcarnitine. Seven of these metabolites were decreased by LPS and increased by metformin, whereas 3-hydroxyoctanoylcarnitine showed the opposite pattern. The shared directional pattern in both compartments suggests that these metabolites may mark a coordinated gut-systemic metabolic response [24,26,27]. More importantly, most of these metabolites were negatively correlated with lung injury indicators, including histological injury score, inflammatory cytokines and immune-cell infiltration markers. This supports their potential relevance to the pulmonary protective phenotype, while not establishing that they originated in the colon or mediated the lung phenotype.

The biological identity of several metformin-responsive metabolites provides plausible mechanistic clues for future studies. Indole-3-acetic acid is a tryptophan-derived metabolite that is often linked to microbial metabolism and mucosal immune regulation [28,29,38,39]. Ferulic acid, 4-hydroxycinnamic acid, 2,6-dihydroxybenzoic acid, and phloroglucinaldehyde are phenolic acid-related molecules that may reflect microbial processing of aromatic compounds and have been associated with antioxidant or anti-inflammatory activity in other contexts [40,41,42]. Pimelic acid and 3-methyladipic acid suggest broader changes in organic acid and lipid-related metabolism, whereas 3-hydroxyoctanoylcarnitine may reflect altered fatty acid oxidation or acylcarnitine handling during inflammatory stress [43]. Together, these metabolites point to a mixed microbial, dietary, and host metabolic signature rather than a single-pathway mechanism.

The metagenomic results further support the involvement of intestinal microbial remodeling. LPS increased richness indices and shifted the overall community structure, while metformin partially reshaped microbial diversity, composition, and predicted gene-content functional potential. Eighteen genera showed an LPS-altered and metformin-reversed pattern, including genera depleted by LPS and increased after metformin treatment as well as genera enriched by LPS and reduced after treatment. This pattern is consistent with previous studies showing that metformin can remodel gut microbiota and microbial functional pathways [15,16,17]. At the functional level, metformin was associated with predicted pathways related to amino acid biosynthesis, pantothenate and CoA biosynthesis, C5-branched dibasic acid metabolism, and protein processing, whereas LPS was linked to pathway labels such as PI3K-Akt signaling, necroptosis, insulin resistance, the sulfur relay system, and teichoic acid biosynthesis. These eukaryotic signaling labels should be interpreted cautiously, because they may reflect homologous gene annotations in shotgun metagenomic data rather than microbial capacity to execute mammalian signaling pathways. Thus, the functional data indicate predicted gene-content potential, not pathway flux, transcription, protein abundance, or enzyme activity.

The integrated multi-omics analysis provides a framework for connecting gut microbial changes, shared metabolites and lung inflammation. The network analysis identified indole-3-acetic acid, 2,6-dihydroxybenzoic acid, and phloroglucinaldehyde as high-degree hub metabolites linking metformin-responsive genera with pulmonary injury indicators. Most nominally significant genus-metabolite correlations were positive, whereas most metabolite-lung injury correlations were negative. This pattern is consistent with an association model in which metformin-responsive microbial–metabolic modules accompany reduced lung inflammation [23,26,32,44]. This is biologically plausible because microbial and host-microbe co-metabolites can enter the circulation and influence distal immune responses, but the present correlation-based analysis cannot resolve directionality, mediation or causation. Direct evidence of metabolite flux, receptor-mediated signaling, or intervention-based rescue will be required to establish causality.

Several limitations should be acknowledged. First, this study used an LPS-induced mouse model, which captures key features of acute inflammatory lung injury but may not fully represent the complexity of human disease or other causes of pulmonary inflammation. In addition, metformin was administered as a seven-day pretreatment before LPS challenge; this preventive protocol is useful for testing biological protection in a preclinical model but does not directly model a clinically deployable post-injury treatment schedule. Second, this study did not include a metformin-only group. Therefore, we cannot distinguish whether some serum, colonic-content metabolite, or gut microbiota changes in the MET (400 mg/kg) group represent normalization of LPS-induced disturbance or metformin-specific effects in otherwise healthy mice. Third, mice were housed three per cage, and housing was not included as a statistical covariate. Therefore, microbiota-related findings could be partially influenced by the effects of the animals’ housing. Fourth, this study mainly establishes associations among metformin treatment, microbial remodeling, metabolite restoration, and lung injury indicators. The correlation networks were based on nominal *p* values and should be considered hypothesis-generating. Fifth, we did not perform AMPK-inhibition, gene-silencing, microbiota-transfer, or metabolite-rescue experiments; thus, the present data do not exclude direct host-intrinsic anti-inflammatory mechanisms of metformin and do not prove that microbiome changes are required for lung protection. Future studies using metformin-only controls, post-LPS therapeutic dosing schedules, antibiotic depletion, fecal microbiota transplantation, germ-free animals, AMPK-pathway perturbation, isotope tracing, targeted metabolomics, and metabolite supplementation will be needed to test the causal structure and clinical translatability of this gut microbiota–metabolite–lung axis.

**Figure 9:**
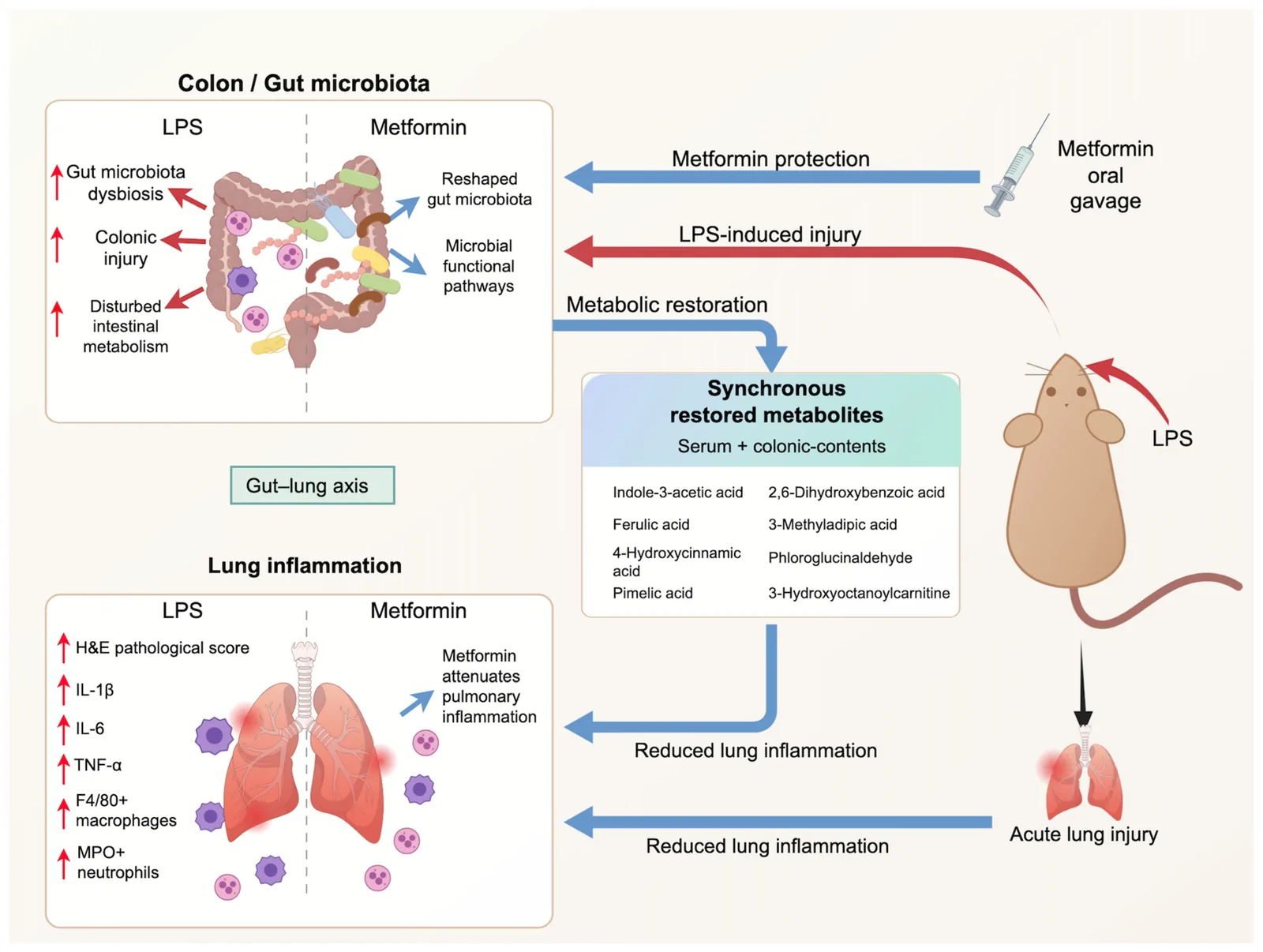
Schematic illustration of the study. Intranasal LPS induced acute pulmonary inflammation, characterized by increased histopathological injury, inflammatory cytokines, and immune-cell infiltration. Metformin gavage was associated with shifts in LPS-disrupted colonic microbiota and predicted microbial functional potential, along with eight metabolites showing synchronized metformin-associated changes in serum and colonic contents. Integrated correlation analyses suggest that metformin-associated microbiota and metabolite remodeling correlated with attenuation of LPS-induced lung injury. The red arrows represent upregulation. Image created with Figdraw platform, with permission.

In conclusion, this study suggests that metformin alleviates LPS-induced pulmonary inflammation and colonic injury in association with coordinated remodeling of the gut microbiota, intestinal luminal metabolites, and circulating metabolites. By integrating pathological assessment, serum and colonic-content metabolomics, and colonic-content metagenomics, we identified shared gut–serum metabolites that correlated with pulmonary inflammatory indices (Figure 9). These findings provide candidate gut-associated and circulating microbial–metabolic signatures and lay a foundation for future mechanistic studies of metformin-mediated protection against LPS-induced inflammatory injury.

## 4. Materials and Methods

### 4.1. Animal Grouping and Administration

Six-week-old SPF male C57BL/6J mice were obtained from GemPharmatech (Nanjing, China). Mice were housed three per cage under specific pathogen-free conditions in a temperature-controlled room (21 ± 2 °C) with a 12 h light/dark cycle and free access to food and sterile drinking water. Following a one-week acclimatization period, mice were randomly assigned to four groups: control (CON), LPS, metformin 200 mg/kg (MET 200 mg/kg), and metformin 400 mg/kg (MET 400 mg/kg). Mice in the MET groups received metformin by oral gavage once daily for 7 days. Mice in the CON and LPS groups received an equal volume of distilled water. On day 7, mice in the LPS and MET groups received a single intranasal administration of LPS (5 mg/kg, dissolved in sterile PBS, Escherichia coli serotype O111:B4, L2630, Sigma Aldrich, Saint Louis, MO, USA) 1 h after the last metformin or PBS administration, whereas CON mice received saline. All animal protocols were reviewed and approved by the Institutional Animal Care and Use Committee of Hebei Medical University (Approval No. IACUC-Hebmu-2026078).

### 4.2. Collection of Samples

At 14 h after LPS challenge, all mice were anesthetized with isoflurane. Orbital blood samples were then collected, and the serum was separated for subsequent biochemical analyses. Following blood collection, the lungs, colonic contents, and colon tissues were harvested from each mouse.

### 4.3. Histopathology and Immunostaining

Lung and colon tissues were fixed in 10% neutral-buffered formalin, embedded in paraffin, sectioned, and stained with hematoxylin and eosin (H&E). Histopathological scoring was performed to evaluate lung injury, following previously established criteria [3]. To assess inflammatory cell infiltration, parallel sections were immunostained with antibodies against F4/80 (Servicebio, Hubei, China) and myeloperoxidase (MPO) (Servicebio, Hubei, China). All stained sections were scanned using a 3D HISTECH system (Budapest, Hungary), and the positive staining areas were quantified using ImageJ software (version 2.3.0). Quantitative analyses were performed on six mice per group (biological replicates) for Figure 1A,B, with five randomly selected fields analyzed per mouse. Images in Figure 2G are representative of six mice per group.

### 4.4. Measurement of Interleukin-1β (IL-1β), Interleukin-6 (IL-6) and Tumor Necrosis Factor-α (TNF-α) in Lung Tissues

Lung tissues were homogenized, and the levels of IL-1β, IL-6, and TNF-α were measured using enzyme-linked immunosorbent assay (ELISA) kits (Liankebio, Hangzhou, China) according to the manufacturer’s instructions.

### 4.5. RT-qPCR Analysis of Inflammatory and Barrier-Related Markers in Colon Tissue

Total RNA was extracted from colon tissue samples using TRIzol (Invitrogen, Carlsbad, CA, USA). First-strand cDNA was synthesized from equal amounts of RNA using the PrimeScript RT Reagent Kit (Takara, Beijing, China). Quantitative real-time PCR (qRT-PCR) was subsequently performed with SYBR Premix Ex Taq II (Takara, Beijing, China) on a QuantStudio Real-Time PCR System (Thermo Fisher Scientific, Waltham, MA, USA). Relative mRNA quantities were determined using the 2-ΔΔCT method. Primer sequences used are listed in Supplementary Table S3.

### 4.6. Metabolomic Analysis

Metabolomic analyses of serum and colonic-content samples were performed using a Thermo Vanquish Flex UPLC system (Thermo Fisher Scientific, Waltham, MA, USA) coupled to a Q Exactive Plus hybrid quadrupole-Orbitrap mass spectrometer (Thermo Fisher Scientific, Waltham, MA, USA). For serum metabolite extraction, 100 μL of serum was mixed with 400 μL of ice-cold methanol and incubated at –20 °C for 30 min for protein precipitation. The samples were then centrifuged at 20,000× *g* for 10 min at 4 °C, and the supernatants were collected for analysis. In the assessment of colonic-content samples, approximately 50 mg of sample was homogenized with 500 μL of 80% ice-cold methanol and incubated at –20 °C for 30 min. After centrifugation at 20,000× *g* for 10 min at 4 °C, the supernatants were collected and centrifuged again for 5 min. Metabolomics was performed on six biological samples per group for CON, LPS, and MET (400 mg/kg), with pooled quality-control (QC) samples prepared by combining equal aliquots of all processed samples to monitor analytical stability.

Partial least-squares discriminant analysis (PLS-DA) and orthogonal partial least-squares discriminant analysis (OPLS-DA) were performed to evaluate group separation and identify metabolites contributing to group discrimination. Volcano plots, heatmaps, metabolite-classification analysis, and Venn diagrams were used to characterize differential and treatment-responsive metabolites. Differential metabolites were screened using the following criteria: nominal *p* < 0.05, fold change >1.2 or <0.83, and VIP value ≥ 1. Kyoto Encyclopedia of Genes and Genomes (KEGG) pathway enrichment analysis was performed using MetaboAnalyst 6.0, and pathways with nominal *p* < 0.05 were considered significantly enriched.

### 4.7. Gut Microbiota Analysis

Colonic-content DNA was extracted using a fecal genomic DNA extraction kit (Tiangen, Beijing, China) according to the manufacturer’s instructions. DNA concentration was measured using the Qubit 1X dsDNA HS Assay Kit (Invitrogen, Carlsbad, CA, USA). For library preparation, 200 ng of qualified DNA was fragmented using a Bioruptor Pico sonicator (Diagenode, Denville, NJ, USA) to an approximate size range of 200–500 bp. Fragment size was assessed using an Agilent 2100 Bioanalyzer with High Sensitivity DNA reagents (5067-4627; Agilent Technologies, Santa Clara, CA, USA). Libraries were prepared using the TruSeq Nano DNA LT Library Preparation Kit (Illumina, San Diego, CA, USA) and quantified using the Qubit assay. Libraries were sequenced by LC-Bio Technology Co., Ltd. (Hangzhou, China) on an Illumina NovaSeq 6000 platform using paired-end 150-bp reads and the NovaSeq 6000 XP 4-Lane Kit v1.5 (300 cycles; 20043131; Illumina). Shotgun metagenomics was performed on six biological samples per group for CON, LPS and MET (400 mg/kg).

Raw FASTQ reads were quality-filtered using fastp (v0.23.4). Host-derived reads were removed by mapping against the mouse reference genome (GRCm39/mm39) using Bowtie2 (v2.2.0). The remaining reads were assembled de novo for each sample using MEGAHIT (v1.2.9). Contigs longer than 500 bp were retained, and coding sequences were predicted using MetaGeneMark (v3.26); predicted CDSs shorter than 100 nt were removed. MMseqs2 (v15-6f452) was used to remove redundancy at 95% sequence identity and 90% coverage, with the longest sequence selected as the representative for each unigene. Bowtie2 was then used to map reads to the unigene set for abundance estimation, and unigenes with no more than two mapped reads in all samples were filtered. DIAMOND (v0.9.14) was used for taxonomic and functional annotation against the NR_meta database and the KEGG (v87.1), GO (2018.12.21), eggNOG (v5.0), PHI (v4.14), CAZy (2022.08.06), CARD (v3.2.5), VFDB (2023.03.03), MGEs (2017.12.28), and BacMet (v2.0) databases. The resulting genus-level taxonomic abundance tables and KEGG pathway-definition abundance tables were used for downstream analyses. QIIME 2 was used for downstream diversity analysis of these abundance tables. Alpha-diversity indices, including observed species, Chao1 richness, and Shannon diversity, were calculated. Beta diversity was evaluated using Bray–Curtis dissimilarity matrices. Principal component analysis (PCA), principal coordinates analysis (PCoA) and non-metric multidimensional scaling (NMDS) were performed in R to visualize differences in overall microbial community structure among the CON, LPS, and MET groups. Genus-level relative abundances were summarized and visualized to compare microbial community composition among groups.

Differentially abundant genera were identified using the Kruskal–Wallis test for comparisons among the three groups, followed by pairwise Wilcoxon rank-sum tests where appropriate. Pairwise *p* values for genus-level comparisons were adjusted using the Benjamini-Hochberg procedure within each analysis family, and adjusted *p* values were used for inferential interpretation. For exploratory screening, nominal *p* < 0.05 and |log2(FC)| > 1 were used as thresholds for differential microbial features; analyses and visualizations were performed in R (v4.2.2). Genera selected for directional response were reported as exploratory when they met nominal pairwise *p* < 0.05 and |log2(FC)| > 1 but did not meet q < 0.05 after adjustment. To investigate associations between the gut microbiota and intestinal metabolites, Spearman correlation analysis was performed between the top 20 abundant genera and the top 20 metformin-responsive colonic metabolites. Correlation coefficients were visualized using heatmaps. Associations with nominal *p* < 0.05 were considered exploratory, with significance levels indicated as * *p* < 0.05, ** *p* < 0.01, and *** *p* < 0.001.

### 4.8. Statistical Analysis

Statistical analyses were performed using GraphPad Prism (version 9.5, San Diego, CA, USA) and R software (version 4.2.2, Vienna, Austria). Lung and colon pathological or inflammatory indices were analyzed using unpaired two-tailed *t*-tests for prespecified pairwise comparisons. Alpha-diversity indices were compared using Wilcoxon rank-sum tests. Differential microbial taxa were assessed using the Kruskal–Wallis test followed by pairwise Wilcoxon rank-sum tests where appropriate, with Benjamini–Hochberg correction applied to the genus-level pairwise comparisons. Metabolomic differences were evaluated using Student’s *t*-test in combination with fold-change and VIP-based screening. Associations among cytokines, differential gut microbial taxa, and metabolites were evaluated using Spearman’s rank correlation analysis across samples. The strength and direction of correlations were described using Spearman’s correlation coefficient and corresponding nominal *p* values. Data were visualized using GraphPad Prism and R software.

## 5. Conclusions

In conclusion, this study suggests that metformin alleviates LPS-induced pulmonary inflammation and colonic injury in association with coordinated remodeling of gut microbiota, intestinal luminal metabolites and circulating metabolites. By integrating pathological assessment, serum and colonic-content metabolomics, and colonic-content metagenomics, we identified shared gut–serum metabolites and metformin-responsive microbial genera that correlated with pulmonary inflammatory indices. These findings provide candidate gut-associated and circulating microbial–metabolic signatures for future mechanistic studies of metformin-mediated protection against LPS-induced inflammatory injury. Because the study did not include a metformin-only group or AMPK-inhibition, gene-silencing, microbiota-transfer, or metabolite-rescue experiments, the present data should be interpreted as identifying associations rather than proving that microbiota–metabolite remodeling is required for lung protection.

## Data Availability

The original contributions presented in this study are included in the article/Supplementary Materials. Further inquiries can be directed to the corresponding author.

## Supplementary Materials

The following supporting information can be downloaded at: https://www.mdpi.com/article/10.3390/ph19101643/s1. Figure S1: Metformin-associated directional shifts in LPS-disrupted colonic-content metabolites. Heatmap showing 112 colonic-content metabolites that met the predefined directional-response criteria in the CON, LPS and MET (400 mg/kg) groups. Metabolites were significantly altered between LPS and CON groups and changed in the opposite direction between MET (400 mg/kg) and LPS groups, with VIP > 1 and nominal *p* < 0.05; metabolites without a significant difference between MET (400 mg/kg) and CON groups were described as shifted toward the control profile.; Figure S2: Genus-level discriminative taxa identified by LEfSe analysis. Genus-level LEfSe analysis of colonic-content metagenomes from the CON, LPS and MET (400 mg/kg) groups. Bars represent linear discriminant analysis (LDA) scores on a log10 scale, and colors indicate the group in which the relative abundance of the corresponding taxon was enriched.; Table S1: Serum shared differential metabolites (n = 218) identified in both the LPS vs. CON and MET vs. LPS comparisons; Table S2: Colonic Content shared differential metabolites (n = 328) identified in both the LPS vs. CON and MET vs. LPS comparisons; Table S3: Primer sequence list for qRT-PCR.

## Abbreviations

The following abbreviations are used in this manuscript:

ALI	Acute lung injury
ARDS	Acute respiratory distress syndrome
ELISA	Enzyme-linked immunosorbent assay
H&E	Hematoxylin and eosin
KEGG	Kyoto Encyclopedia of Genes and Genomes
LPS	Lipopolysaccharide
MET	Metformin
OPLS-DA	Orthogonal partial least-squares discriminant analysis
QC	Quality control
RT-qPCR	Reverse-transcription quantitative PCR
VIP	Variable importance in projection

## References

[1] Matthay M.A., Zemans R.L., Zimmerman G.A., Arabi Y.M., Beitler J.R., Mercat A., Herridge M., Randolph A.G., Calfee C.S. (2019). Acute Respiratory Distress Syndrome. Nat. Rev. Dis. Primers.

[2] Meyer N.J., Gattinoni L., Calfee C.S. (2021). Acute Respiratory Distress Syndrome. Lancet.

[3] Matute-Bello G., Downey G., Moore B.B., Groshong S.D., Matthay M.A., Slutsky A.S., Kuebler W.M., Acute Lung Injury in Animals Study Group (2011). An Official American Thoracic Society Workshop Report: Features and Measurements of Experimental Acute Lung Injury in Animals. Am. J. Respir. Cell Mol. Biol..

[4] Kulkarni H.S., Lee J.S., Bastarache J.A., Kuebler W.M., Downey G.P., Albaiceta G.M., Altemeier W.A., Artigas A., Bates J.H.T., Calfee C.S. (2022). Update on the Features and Measurements of Experimental Acute Lung Injury in Animals: An Official American Thoracic Society Workshop Report. Am. J. Respir. Cell Mol. Biol..

[5] Özçam M., Lynch S.V. (2024). The Gut-Airway Microbiome Axis in Health and Respiratory Diseases. Nat. Rev. Microbiol..

[6] Budden K.F., Gellatly S.L., Wood D.L.A., Cooper M.A., Morrison M., Hugenholtz P., Hansbro P.M. (2017). Emerging Pathogenic Links between Microbiota and the Gut-Lung Axis. Nat. Rev. Microbiol..

[7] Enaud R., Prevel R., Ciarlo E., Beaufils F., Wieërs G., Guery B., Delhaes L. (2020). The Gut-Lung Axis in Health and Respiratory Diseases: A Place for Inter-Organ and Inter-Kingdom Crosstalks. Front. Cell. Infect. Microbiol..

[8] Dang A.T., Marsland B.J. (2019). Microbes, Metabolites, and the Gut-Lung Axis. Mucosal Immunol..

[9] Bailey C.J. (2017). Metformin: Historical Overview. Diabetologia.

[10] Rena G., Hardie D.G., Pearson E.R. (2017). The Mechanisms of Action of Metformin. Diabetologia.

[11] Foretz M., Guigas B., Viollet B. (2023). Metformin: Update on Mechanisms of Action and Repurposing Potential. Nat. Rev. Endocrinol..

[12] Kristófi R., Eriksson J.W. (2021). Metformin as an Anti-Inflammatory Agent: A Short Review. J. Endocrinol..

[13] Viollet B., Guigas B., Sanz Garcia N., Leclerc J., Foretz M., Andreelli F. (2012). Cellular and Molecular Mechanisms of Metformin: An Overview. Clin. Sci..

[14] McCreight L.J., Bailey C.J., Pearson E.R. (2016). Metformin and the Gastrointestinal Tract. Diabetologia.

[15] Wu H., Esteve E., Tremaroli V., Khan M.T., Caesar R., Mannerås-Holm L., Ståhlman M., Olsson L.M., Serino M., Planas-Fèlix M. (2017). Metformin Alters the Gut Microbiome of Individuals with Treatment-Naive Type 2 Diabetes, Contributing to the Therapeutic Effects of the Drug. Nat. Med..

[16] Forslund K., Hildebrand F., Nielsen T., Falony G., Le Chatelier E., Sunagawa S., Prifti E., Vieira-Silva S., Gudmundsdottir V., Pedersen H.K. (2015). Disentangling Type 2 Diabetes and Metformin Treatment Signatures in the Human Gut Microbiota. Nature.

[17] Sun L., Xie C., Wang G., Wu Y., Wu Q., Wang X., Liu J., Deng Y., Xia J., Chen B. (2018). Gut Microbiota and Intestinal FXR Mediate the Clinical Benefits of Metformin. Nat. Med..

[18] Lee H., Ko G. (2014). Effect of Metformin on Metabolic Improvement and Gut Microbiota. Appl. Environ. Microbiol..

[19] Xian H., Liu Y., Rundberg Nilsson A., Gatchalian R., Crother T.R., Tourtellotte W.G., Zhang Y., Aleman-Muench G.R., Lewis G., Chen W. (2021). Metformin Inhibition of Mitochondrial ATP and DNA Synthesis Abrogates NLRP3 Inflammasome Activation and Pulmonary Inflammation. Immunity.

[20] Wang Y., Chen H., Sun C., Shen H., Cui X. (2021). Metformin Attenuates Lipopolysaccharide-Induced Epithelial Cell Senescence by Activating Autophagy. Cell Biol. Int..

[21] Wu K., Tian R., Huang J., Yang Y., Dai J., Jiang R., Zhang L. (2018). Metformin Alleviated Endotoxemia-Induced Acute Lung Injury via Restoring AMPK-Dependent Suppression of mTOR. Chem.-Biol. Interact..

[22] Cheng M., Ren L., Jia X., Wang J., Cong B. (2024). Understanding the Action Mechanisms of Metformin in the Gastrointestinal Tract. Front. Pharmacol..

[23] Rooks M.G., Garrett W.S. (2016). Gut Microbiota, Metabolites and Host Immunity. Nat. Rev. Immunol..

[24] Postler T.S., Ghosh S. (2017). Understanding the Holobiont: How Microbial Metabolites Affect Human Health and Shape the Immune System. Cell Metab..

[25] Levy M., Kolodziejczyk A.A., Thaiss C.A., Elinav E. (2017). Dysbiosis and the Immune System. Nat. Rev. Immunol..

[26] Blander J.M., Longman R.S., Iliev I.D., Sonnenberg G.F., Artis D. (2017). Regulation of Inflammation by Microbiota Interactions with the Host. Nat. Immunol..

[27] Krautkramer K.A., Fan J., Bäckhed F. (2021). Gut Microbial Metabolites as Multi-Kingdom Intermediates. Nat. Rev. Microbiol..

[28] Agus A., Planchais J., Sokol H. (2018). Gut Microbiota Regulation of Tryptophan Metabolism in Health and Disease. Cell Host Microbe.

[29] Zelante T., Iannitti R.G., Cunha C., De Luca A., Giovannini G., Pieraccini G., Zecchi R., D’Angelo C., Massi-Benedetti C., Fallarino F. (2013). Tryptophan Catabolites from Microbiota Engage Aryl Hydrocarbon Receptor and Balance Mucosal Reactivity via Interleukin-22. Immunity.

[30] Hang S., Paik D., Yao L., Kim E., Trinath J., Lu J., Ha S., Nelson B.N., Kelly S.P., Wu L. (2019). Bile Acid Metabolites Control TH17 and Treg Cell Differentiation. Nature.

[31] Fiorucci S., Biagioli M., Zampella A., Distrutti E. (2018). Bile Acids Activated Receptors Regulate Innate Immunity. Front. Immunol..

[32] Trompette A., Gollwitzer E.S., Yadava K., Sichelstiel A.K., Sprenger N., Ngom-Bru C., Blanchard C., Junt T., Nicod L.P., Harris N.L. (2014). Gut Microbiota Metabolism of Dietary Fiber Influences Allergic Airway Disease and Hematopoiesis. Nat. Med..

[33] Wahlström A., Sayin S.I., Marschall H.-U., Bäckhed F. (2016). Intestinal Crosstalk between Bile Acids and Microbiota and Its Impact on Host Metabolism. Cell Metab..

[34] Dalile B., Van Oudenhove L., Vervliet B., Verbeke K. (2019). The Role of Short-Chain Fatty Acids in Microbiota-Gut-Brain Communication. Nat. Rev. Gastroenterol. Hepatol..

[35] O’Neill L.A.J., Kishton R.J., Rathmell J. (2016). A Guide to Immunometabolism for Immunologists. Nat. Rev. Immunol..

[36] Tannahill G.M., Curtis A.M., Adamik J., Palsson-McDermott E.M., McGettrick A.F., Goel G., Frezza C., Bernard N.J., Kelly B., Foley N.H. (2013). Succinate Is an Inflammatory Signal That Induces IL-1β through HIF-1α. Nature.

[37] Mills E.L., Kelly B., Logan A., Costa A.S.H., Varma M., Bryant C.E., Tourlomousis P., Däbritz J.H.M., Gottlieb E., Latorre I. (2016). Succinate Dehydrogenase Supports Metabolic Repurposing of Mitochondria to Drive Inflammatory Macrophages. Cell.

[38] Bansal T., Alaniz R.C., Wood T.K., Jayaraman A. (2010). The Bacterial Signal Indole Increases Epithelial-Cell Tight-Junction Resistance and Attenuates Indicators of Inflammation. Proc. Natl. Acad. Sci. USA.

[39] Roager H.M., Licht T.R. (2018). Microbial Tryptophan Catabolites in Health and Disease. Nat. Commun..

[40] Dong X., Huang R. (2022). Ferulic Acid: An Extraordinarily Neuroprotective Phenolic Acid with Anti-Depressive Properties. Phytomedicine.

[41] Cardona F., Andrés-Lacueva C., Tulipani S., Tinahones F.J., Queipo-Ortuño M.I. (2013). Benefits of Polyphenols on Gut Microbiota and Implications in Human Health. J. Nutr. Biochem..

[42] Selma M.V., Espín J.C., Tomás-Barberán F.A. (2009). Interaction between Phenolics and Gut Microbiota: Role in Human Health. J. Agric. Food Chem..

[43] McCoin C.S., Knotts T.A., Adams S.H. (2015). Acylcarnitines--Old Actors Auditioning for New Roles in Metabolic Physiology. Nat. Rev. Endocrinol..

[44] Schuijt T.J., Lankelma J.M., Scicluna B.P., de Sousa e Melo F., Roelofs J.J.T.H., de Boer J.D., Hoogendijk A.J., de Beer R., de Vos A., Belzer C. (2016). The Gut Microbiota Plays a Protective Role in the Host Defence against Pneumococcal Pneumonia. Gut.

