# Supplementary material for: Multi-Omics Analysis Identifies Gut-Associated Metabolic Signatures Associated with Metformin-Mediated Protection Against LPS-Induced Lung Injury
Source: Pharmaceuticals (Basel). 2026 Oct 18;19(10):1643. doi: 10.3390/ph19101643 (PMC13708650; doi:10.3390/ph19101643)
Supplement: Supplementary file 1 [file pharmaceuticals-19-01643-s001.zip › Supplementary Figures.pdf]

# Supplementary Figures

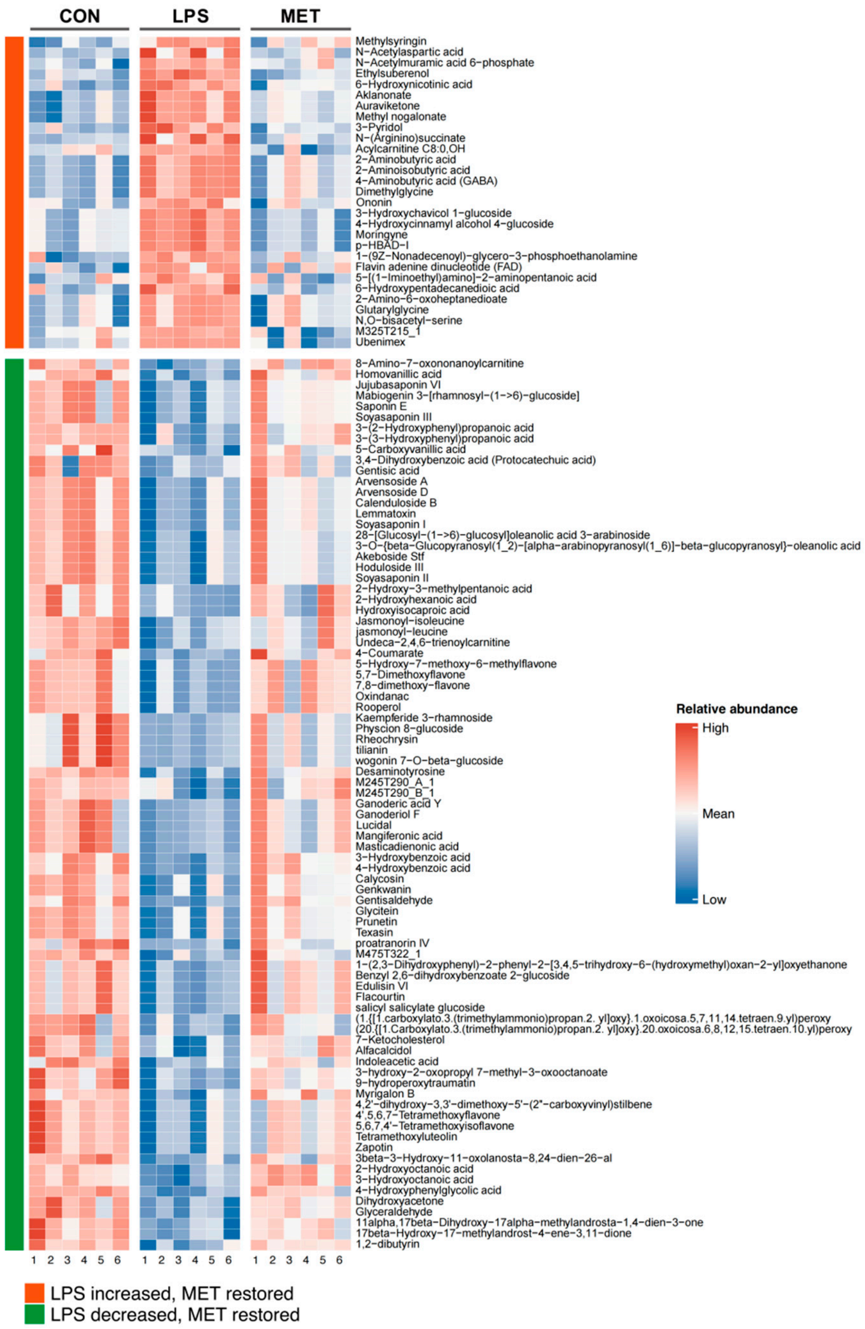


Supplementary Figure S1. Metformin-associated directional shifts in LPS-disrupted colonic-content metabolites. Heatmap showing 112 colonic-content metabolites that met the predefined directional-response criteria in the CON, LPS and MET (400 mg/kg) groups. Metabolites were significantly altered between LPS and CON groups and changed in the opposite direction between MET (400 mg/kg) and LPS groups, with VIP＞1 and nominal $P$＜0.05; metabolites without a significant difference between MET (400 mg/kg) and CON groups were described as shifted toward the control profile.

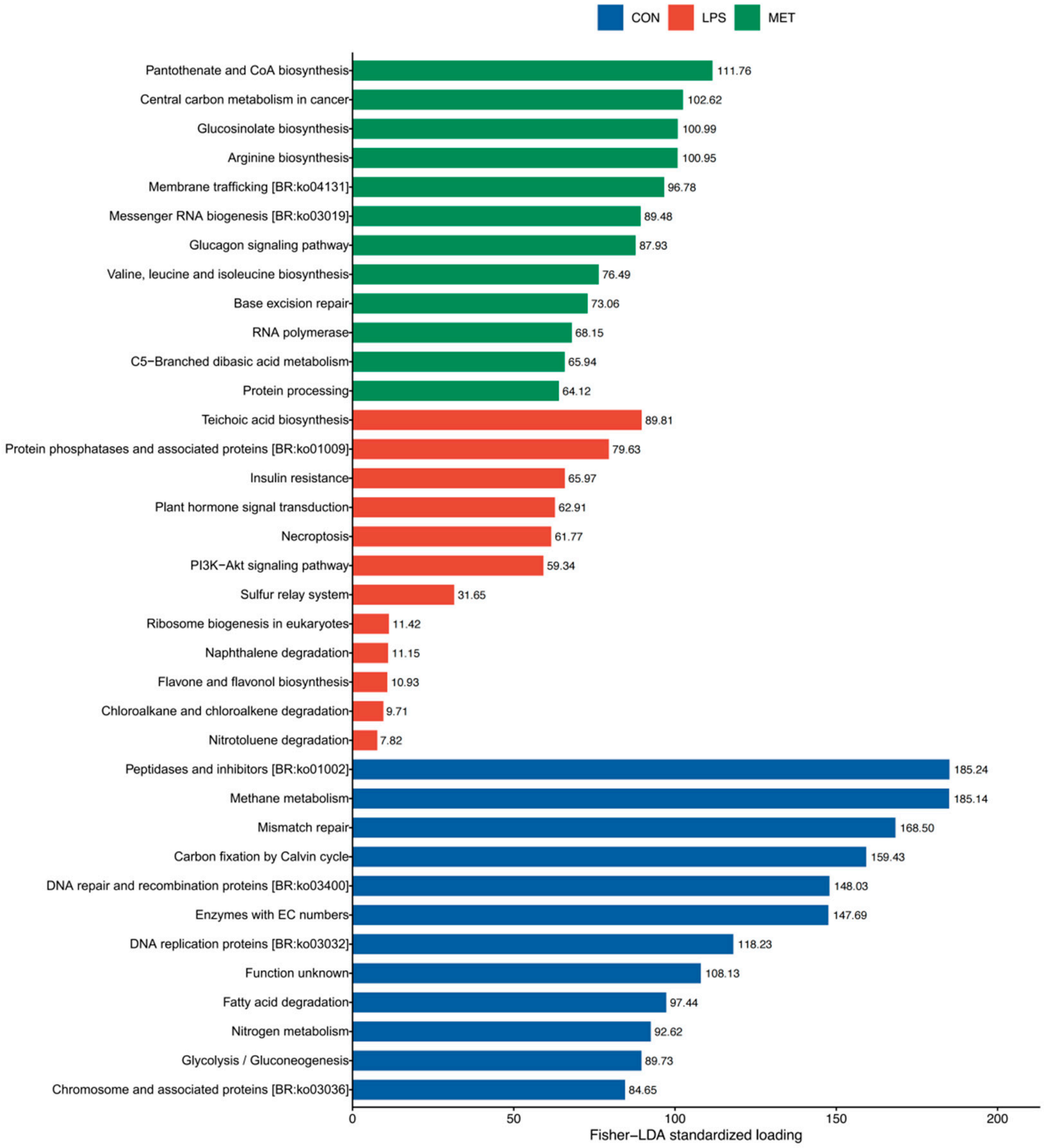


Supplementary Figure S2. Genus-level discriminative taxa identified by LEfSe analysis. Genus-level LEfSe analysis of colonic-content metagenomes from the CON, LPS and MET (400 mg/kg) groups. Bars represent linear discriminant analysis (LDA) scores on a log10 scale, and colours indicate the group in which the relative abundance of the corresponding taxon was enriched.
