# Supplementary material for: Multi-Omics Analysis Identifies Gut-Associated Metabolic Signatures Associated with Metformin-Mediated Protection Against LPS-Induced Lung Injury
Source: Pharmaceuticals (Basel). 2026 Oct 18;19(10):1643. doi: 10.3390/ph19101643 (PMC13708650; doi:10.3390/ph19101643)
Supplement: Supplementary file 1 [file pharmaceuticals-19-01643-s001.zip › Supplementary Tables.pdf]

Supplementary Tables

Supplementary Table S1. Serum shared differential metabolites (n = 218) identified in both the LPS vs CON and MET vs LPS comparisons

Feature ID	Name	LPS vs CON P	LPS vs CON VIP	MET vs LPS P	MET vs LPS VIP	HMDB	KEGG	Class	LPS vs CON	MET vs LPS
M319T411_E_1	(ent-15beta)-15-Hydroxy-19-trachylobanoic acid	7.74E-4	1.222	0.0028	1.741	HMDB0036839	NA	Prenol lipids	up	down
M301T400_B_1	1,1,1-Tri(butyryloxy)propane	9.94E-6	1.862	0.017	1.597	HMDB0257803	NA	Carboxylic acids and derivatives	down	up
M225T429_2_A_1	1,1-Diethoxy-3,7-dimethyl-2,6-octadiene	1.49E-6	1.622	0.0132	1.441	HMDB0040228	NA	Prenol lipids	up	down
M183T385_B_1	1-Acetylcyclohexyl acetate	2.04E-6	1.931	0.0012	2.202	HMDB0039710	NA	Organooxygen compounds	down	up
M257T408_B_1	1-Hydroxypropan-2-yl 2-isopropyl-5-methylcyclohexyl carbonate	2.88E-7	1.625	0.0059	1.634	HMDB0059947	NA	Prenol lipids	down	up
M193T413_E_1	1-Phenylethyl butyrate	1.05E-4	1.541	0.0125	1.419	HMDB0037717	NA	Benzene and substituted derivatives	down	up
M450T491_1	1-palmitoleoyl-sn-glycero-3-phosphoethanolamine	2.63E-6	1.258	0.0064	1.219	NA	NA	NA	down	up
M309T484_A_1	10-Hydroperoxyoctadeca-8,12,15-trienoate	1.05E-4	1.368	0.0406	1.085	NA	C20704	NA	down	up

M183T385_C_1	10-Oxo-8-decenoic acid	2.04E-6	1.931	0.0012	2.202	HMDB0040883	NA	Fatty Acyls	down	up
M185T414_2_C_1	10-Oxodecanoate	1.18E-6	1.873	3.57E-4	2.212	NA	C02217	NA	down	up
M247T496_C_1	10-phenyldecanoate	1.67E-7	1.578	0.0019	1.516	NA	NA	NA	up	down
M335T411_1	11-Deoxyprostaglandin E2	0.003	1.079	0.0045	1.633	NA	NA	NA	up	down
M343T475_D_1	11alpha-Hydroxy-12alpha-methyl-pregn-4-ene-3,20-dione	5.33E-5	1.149	0.0074	1.196	NA	C14964	NA	up	down
M363T363_1	11beta,21-Dihydroxy-3,20-oxo-5beta-pregnan-18-al	0.0299	1.232	0.0296	1.075	NA	C05473	NA	up	down
M225T429_2_B_1	14-Methyloxacyclotetradecan-2-one	1.49E-6	1.622	0.0132	1.441	HMDB0030921	NA	Macrolides and analogues	up	down
M271T476_1	16-Hydroxypalmitic acid	0.0011	1.134	0.0378	1.274	HMDB0006294	C18218	Fatty Acyls	up	down
M391T386_1	17alpha-Hydroxypregnenolone	2.64E-5	2.055	0.0174	1.802	HMDB0000363	C05138	Steroids and steroid derivatives	down	up
M319T411_B_1	17beta-Hydroxy-16alpha-methoxy-androst-4-en-3-one	7.74E-4	1.222	0.0028	1.741	NA	C14929	NA	up	down
M319T424_1	17beta-Methoxyandrost-5-ene-3beta,16beta-diol	2.67E-4	1.870	0.004	2.161	NA	C15138	NA	down	up
M181T341_	2',4'-Dihydroxy-6'-methox	9.01E-4	1.979	0.0292	1.962	HMDB0041270	NA	Organooxygen	down	up

1	yacetophenone							compounds		
M237T343_1	2,3-Dihydroxypropyl 4-phenylbutanoate	7.81E-6	2.288	6.94E-4	2.572	HMDB0257749	NA	Glycerolipids	down	up
M327T404_1	2,3-Dinor-8-iso prostaglandin F1alpha	1.48E-8	1.733	0.0263	1.436	NA	C14795	NA	down	up
M417T422_1	2,3-di-isovaleroyl-4-isobutanoyl-glucose	1.79E-5	1.480	0.0085	1.231	NA	NA	NA	down	up
M165T416_A_1	2,4,7-Decatrienoic acid	2.02E-7	2.112	0.0105	1.370	HMDB0035235	NA	Fatty Acyls	up	down
M153T307_B_1	2,6-Dihydroxybenzoic acid	1.06E-4	2.063	0.02	1.828	HMDB0013676	C21298	Benzene and substituted derivatives	down	up
M165T416_E_1	2-Butylhydroquinone	2.02E-7	2.112	0.0105	1.370	HMDB0245044	NA	Phenols	up	down
M249T444_A_1	2-Ethylhexyl-4-hydroxybenzoate	1.79E-8	1.508	0.0057	1.150	NA	C14716	NA	down	up
M187T437_1	2-Hydroxydecanoic acid	2.90E-8	1.348	8.66E-4	1.611	HMDB0094656	NA	Fatty Acyls	down	up
M159T354_B_1	2-Hydroxyoctanoic acid	7.90E-5	1.095	0.0174	1.084	HMDB0000711	NA	Fatty Acyls	up	down
M257T408_C_1	2-Hydroxypropyl 2-isopropyl-5-methylcyclohexyl carbonate	2.88E-7	1.625	0.0059	1.634	HMDB0059946	NA	Prenol lipids	down	up
M185T414_2_A_1	2-Oxodecanoic acid	1.18E-6	1.873	3.57E-4	2.212	NA	C13945	NA	down	up

M157T384_1	2-Oxooctanoic acid	1.06E-4	1.094	0.0013	1.435	HMDB0013211	NA	Keto acids and derivatives	down	up
M247T496_B_1	2-Phenylethyl octanoate	1.67E-7	1.578	0.0019	1.516	HMDB0037719	NA	Fatty Acyls	up	down
M243T527_1	2S-Hydroxytetradecanoic acid	4.42E-5	1.502	0.0031	1.361	HMDB0002261	C13790	Fatty Acyls	up	down
M317T281_C_1	3,3',4',5,5',8-Hexahydroxyflavone	1.98E-5	1.998	0.0283	1.387	HMDB0037357	NA	Flavonoids	down	up
M317T281_E_1	3,4',5,6,7,8-Hexahydroxyflavone	1.98E-5	1.998	0.0283	1.387	HMDB0303817	NA	Flavonoids	down	up
M345T495_A_1	3,4-Dihydro-8-hydroxy-3-tridecyl-1H-2-benzopyran-1-one	2.57E-5	1.448	0.0452	1.176	HMDB0041302	NA	Benzopyrans	up	down
M302T237_1	3-Hydroxy-cis-5-octenoyl carnitine	1.67E-5	1.408	0.0474	1.127	HMDB0240766	NA	Fatty Acyls	up	down
M215T462_1	3-Hydroxylauric acid	7.44E-6	1.405	0.0196	1.454	HMDB0000387	NA	Hydroxy acids and derivatives	up	down
M243T501_1	3-Hydroxymyristic acid	2.58E-5	1.483	0.026	1.455	HMDB0010731	C22001	Fatty Acyls	up	down
M159T354_A_1	3-Hydroxyoctanoic acid	7.90E-5	1.095	0.0174	1.084	HMDB0001954	C20793	Hydroxy acids and derivatives	up	down
M273T361_1	3-Hydroxytetradecanedioic acid	7.70E-7	2.101	4.78E-4	2.459	HMDB0000394	NA	Fatty Acyls	down	up
M186T344_A_1	3-Indoleacrylic acid	5.55E-4	1.815	0.0457	1.628	HMDB0000734	C21283	Indoles and derivatives	down	up

M281T402_C_1	3-Methyl-5-pentyl-2-furanheptanoic acid	8.81E-7	1.160	1.39E-4	1.161	HMDB0112095	NA	Fatty Acyls	down	up
M281T402_A_1	3-Methyl-5-propyl-2-furannonanoic acid	8.81E-7	1.160	1.39E-4	1.161	HMDB0094642	NA	Fatty Acyls	down	up
M159T248_B_1	3-Methyladipic acid	2.64E-6	1.296	0.0039	1.229	HMDB0000555	NA	Fatty Acyls	down	up
M290T71_1	3-Methylglutarylcarnitine (C6:0,COOH,Me)	3.92E-6	1.348	0.0021	1.658	HMDB0000552	NA	Fatty Acyls	up	down
M185T414_2_B_1	3-Oxodecanoate	1.18E-6	1.873	3.57E-4	2.212	HMDB0010724	C20714	Keto acids and derivatives	down	up
M213T440_1	3-Oxododecanoic acid	2.37E-8	1.780	0.0221	1.618	HMDB0010727	C02367	Keto acids and derivatives	up	down
M327T487_A_1	3-undecyl-2-hydroxyl-1,4-naphthoquinone	9.24E-6	1.388	0.0322	1.339	NA	NA	NA	up	down
M249T516_B_1	4,7,10,13-Hexadecatetraenoic acid	2.25E-6	1.757	0.0038	1.586	HMDB0246620	NA	Fatty Acyls	up	down
M165T416_C_1	4-Butoxyphenol	2.02E-7	2.112	0.0105	1.370	NA	C14351	NA	up	down
M165T416_D_1	4-Butylresorcinol	2.02E-7	2.112	0.0105	1.370	NA	C18343	NA	up	down
M163T295_A_1	4-Hydroxycinnamic acid	0.0023	1.252	1.31E-4	2.932	HMDB0002035	C00811	Cinnamic acids and derivatives	down	up
M193T413_B_1	4-Methylphenyl 3-methylbutanoate	1.05E-4	1.541	0.0125	1.419	HMDB0037709	NA	Phenol esters	down	up
M387T351_	5,6-Dihydroxyprostagland	0.0139	1.022	0.0376	1.493	HMDB0012109	NA	Fatty Acyls	up	down

1	in F1a									
M301T473_C_1	5,8,11-Eicosatriynoic acid	5.83E-7	1.468	0.0437	1.248	HMDB0246870	NA	Fatty Acyls	up	down
M281T402_B_1	5-Heptyl-3-methyl-2-furanpentanoic acid	8.81E-7	1.160	1.39E-4	1.161	HMDB0112087	NA	Fatty Acyls	down	up
M281T402_D_1	5-Pentyl-2-furanoctanoic acid	8.81E-7	1.160	1.39E-4	1.161	HMDB0112098	NA	Fatty Acyls	down	up
M447T310_B_1	6-Hydroxyluteolin 7-rhamnoside	5.79E-4	2.075	0.0039	2.794	HMDB0303814	NA	Flavonoids	down	up
M327T487_B_1	6-Methylene-4-pregnene-3,20-dione	9.24E-6	1.388	0.0322	1.339	HMDB0247087	NA	Steroids and steroid derivatives	up	down
M255T338_1	7,8-Dihydroxyflavone	1.71E-5	2.318	2.01E-4	3.063	HMDB0247297	NA	Flavonoids	down	up
M309T484_C_1	9HPOT	1.05E-4	1.368	0.0406	1.085	NA	C16321	NA	down	up
M332T342_1	Acylcarnitine C10:0,OH	1.53E-5	1.404	0.0088	1.477	NA	NA	NA	up	down
M310T324_1	Acylcarnitine C10:3	5.56E-5	2.391	0.0159	1.562	HMDB0240752	NA	Fatty Acyls	up	down
M360T386_1	Acylcarnitine C12:0,OH	2.14E-6	1.344	0.0057	1.587	NA	NA	NA	up	down
M342T392_1	Acylcarnitine C12:1	5.96E-6	1.654	0.0097	1.654	HMDB0241215	NA	Fatty Acyls	up	down
M356T414_1	Acylcarnitine C13:1	1.23E-4	1.769	0.0109	1.645	HMDB0241313	NA	Fatty Acyls	up	down

M386T408_A_1	Acylcarnitine C14:1,OH	6.47E-7	1.617	0.0102	1.527	NA	NA	NA	up	down
M368T408_1	Acylcarnitine C14:2	2.68E-6	1.709	0.0121	1.571	HMDB0241377	NA	Fatty Acyls	up	down
M384T388_1	Acylcarnitine C14:2,OH	1.99E-7	1.705	0.0027	1.751	NA	NA	NA	up	down
M366T388_1	Acylcarnitine C14:3	3.67E-4	1.929	0.0217	1.718	NA	NA	NA	up	down
M430T389_1	Acylcarnitine C16:0,COOH	3.32E-4	1.012	0.0052	1.164	HMDB0000712	NA	Fatty Acyls	up	down
M398T458_1	Acylcarnitine C16:0,OH	5.72E-5	1.511	0.0185	1.344	NA	NA	NA	up	down
M398T474_1	Acylcarnitine C16:1	4.48E-6	1.455	0.0448	1.161	HMDB0006317	NA	Fatty Acyls	up	down
M394T426_1	Acylcarnitine C16:2,OH	3.86E-7	1.808	0.0123	1.394	NA	NA	NA	up	down
M412T473_1	Acylcarnitine C17:1	0.0045	1.764	0.0113	1.334	NA	NA	NA	up	down
M440T449_1	Acylcarnitine C18:0,COOH	2.22E-7	1.725	0.028	1.389	NA	NA	NA	up	down
M424T481_A_1	Acylcarnitine C18:2	1.84E-4	1.715	0.0341	1.328	HMDB0241541	NA	Fatty Acyls	up	down
M466T462_1	Acylcarnitine C20:3,OH	2.01E-4	2.086	0.023	1.073	NA	NA	NA	up	down
M464T451_	Acylcarnitine C20:4,OH	2.89E-8	1.829	0.0217	1.423	NA	NA	NA	up	down

1										
M304T278_1	Acylcarnitine C8:0,OH	1.78E-4	1.398	0.0034	1.472	NA	NA	NA	up	down
M286T301_1	Acylcarnitine C8:1	2.88E-4	1.794	0.0035	1.412	HMDB0013324	NA	Fatty Acyls	up	down
M316T216_1	Acylcarnitine C8:1,COOH	3.07E-4	1.424	0.0393	1.459	HMDB0241710	NA	Fatty Acyls	up	down
M445T296_A_1	Apigenin 7-glucuronide	1.74E-5	2.437	4.83E-4	3.099	HMDB0240480	NA	Flavonoids	down	up
M448T464_1	Arachidonoylcarnitine (C20:4)	3.35E-7	1.445	0.0017	1.242	HMDB0006455	NA	Fatty Acyls	up	down
M187T328_1	Azelaic acid	8.01E-6	1.173	0.0018	1.372	HMDB0000784	C08261	Fatty Acyls	down	up
M445T296_B_1	Baicalin	1.74E-5	2.437	4.83E-4	3.099	HMDB0041832	C10025	Flavonoids	down	up
M193T413_D_1	Benzyl 2-methylbutanoate	1.05E-4	1.541	0.0125	1.419	HMDB0303579	NA	Benzene and substituted derivatives	down	up
M319T411_D_1	Ciliaric acid	7.74E-4	1.222	0.0028	1.741	HMDB0302679	NA	Prenol lipids	up	down
M301T473_B_1	Cosahexaenoic acid	5.83E-7	1.468	0.0437	1.248	HMDB0250481	NA	Fatty Acyls	up	down
M321T462_1	Cytosporone B	3.05E-6	1.608	0.0359	1.426	HMDB0250727	NA	Organooxygen compounds	up	down
M231T280_	Dazoxiben	4.11E-6	1.634	0.0058	1.431	HMDB0250886	NA	Benzene and	down	up

1								substituted derivatives		
M447T310_C_1	Delphinidin 3-rhamnoside [M]	5.79E-4	2.075	0.0039	2.794	HMDB0303800	NA	Flavonoids	down	up
M257T408_A_1	Dibutyl adipate	2.88E-7	1.625	0.0059	1.634	HMDB0251172	C14253	Fatty Acyls	down	up
M345T495_D_1	Dihydrotestosterone propionate	2.57E-5	1.448	0.0452	1.176	HMDB0251111	NA	Steroids and steroid derivatives	up	down
M229T385_1	Diisopropyl adipate	2.41E-6	1.262	0.0072	1.362	HMDB0041613	C14523	Fatty Acyls	down	up
M247T496_A_1	Dimethylbenzyl carbinyl hexanoate(M247T496_A_1)	1.67E-7	1.578	0.0019	1.516	HMDB0032228	NA	Benzene and substituted derivatives	up	down
M249T431_A_1	Dimethylbenzyl carbinyl hexanoate(M249T431_A_1)	7.62E-5	1.451	0.0158	1.406	HMDB0032228	NA	Benzene and substituted derivatives	down	up
M895T595_1	Dioleoylphosphatidyl-ddc	0.0019	1.108	0.0426	1.188	HMDB0251429	NA	Glycerophospholipids	down	up
M327T540_4_1	Docosahexaenoic acid (DHA)	7.05E-7	1.259	0.0137	1.010	HMDB0002183	C06429	Fatty Acyls	up	down
M329T549_2_1	Docosapentaenoic acid (DPA)	1.07E-6	1.404	0.0175	1.130	HMDB0006528	C16513	Fatty Acyls	up	down
M199T511_2_1	Dodecanoic acid	4.10E-5	1.137	0.0225	1.185	HMDB0000638	C02679	Fatty Acyls	up	down
M433T340_	Eriodictin	0.0013	2.142	0.0184	2.511	HMDB0037480	NA	Flavonoids	down	up

C_1										
M249T444_B_1	Ethylhexyl salicylate	1.79E-8	1.508	0.0057	1.150	HMDB0061839	NA	Benzene and substituted derivatives	down	up
M373T330_1	ExMrn00001160	1.88E-4	2.182	0.0014	3.122	NA	NA	NA	down	up
M369T225_1	ExMrn00022965	1.04E-4	2.218	6.26E-4	3.111	NA	NA	NA	down	up
M273T384_A_1	ExMrn00023318	0.0018	1.470	0.0058	2.217	NA	NA	NA	down	up
M445T460_D_1	ExMrn00043144	2.26E-6	1.138	0.0071	1.038	NA	NA	NA	down	up
M509T400_A_1	ExMrn00051573	0.006	1.852	0.0076	2.811	NA	NA	NA	down	up
M509T400_B_1	ExMrn00053272	0.006	1.852	0.0076	2.811	NA	NA	NA	down	up
M375T219_C_1	ExMrn00066987	0.0307	1.022	0.0283	1.202	NA	NA	NA	up	up
M375T219_E_1	ExMrn00067021	0.0307	1.022	0.0283	1.202	NA	NA	NA	up	up
M375T219_A_1	ExMrn00067060	0.0307	1.022	0.0283	1.202	NA	NA	NA	up	up
M375T219_B_1	ExMrn00067323	0.0307	1.022	0.0283	1.202	NA	NA	NA	up	up
M375T219_	ExMrn00067337	0.0307	1.022	0.0283	1.202	NA	NA	NA	up	up

D_1										
M445T460_A_1	ExMrn00071053	2.26E-6	1.138	0.0071	1.038	NA	NA	NA	down	up
M445T460_B_1	ExMrn00071064	2.26E-6	1.138	0.0071	1.038	NA	NA	NA	down	up
M445T460_C_1	ExMrn00071070	2.26E-6	1.138	0.0071	1.038	NA	NA	NA	down	up
M273T384_B_1	ExMrn00073600	0.0018	1.470	0.0058	2.217	NA	NA	NA	down	up
M273T384_C_1	ExMrn00073601	0.0018	1.470	0.0058	2.217	NA	NA	NA	down	up
M462T376_A_1	ExMrn00095867	8.89E-4	1.822	0.0358	1.970	NA	NA	NA	down	up
M462T376_C_1	ExMrn00096079	8.89E-4	1.822	0.0358	1.970	NA	NA	NA	down	up
M462T376_B_1	ExMrn00096619	8.89E-4	1.822	0.0358	1.970	NA	NA	NA	down	up
M445T460_E_1	ExMrn00101301	2.26E-6	1.138	0.0071	1.038	NA	NA	NA	down	up
M509T400_D_1	ExMrn00119201	0.006	1.852	0.0076	2.811	NA	NA	NA	down	up
M509T400_E_1	ExMrn00119202	0.006	1.852	0.0076	2.811	NA	NA	NA	down	up
M509T400_C_1	ExMrn00119203	0.006	1.852	0.0076	2.811	NA	NA	NA	down	up

M487T278_1	ExMrn00123160	5.33E-4	1.173	0.0054	1.807	NA	NA	NA	up	down
M193T293_1	Ferulic acid	2.42E-6	2.110	1.10E-5	2.918	HMDB0000954	C01494	Cinnamic acids and derivatives	down	up
M273T265_A_1	Ferulic acid 4-O-sulfate	2.47E-6	2.022	5.71E-5	2.932	HMDB0029200	NA	Cinnamic acids and derivatives	down	up
M425T416_1	Gemfibrozil 1-O-beta-Glucuronide	4.03E-5	2.175	0.0063	2.036	HMDB0247652	NA	Organooxygen compounds	down	up
M237T438_B_1	Geranyl acetoacetate	6.22E-6	1.101	0.0038	1.397	HMDB0038256	NA	Fatty Acyls	up	down
M345T495_C_1	Ginkgoic acid	2.57E-5	1.448	0.0452	1.176	HMDB0033897	C10794	Benzene and substituted derivatives	up	down
M327T518_A_1	Glyceryl palmitoleate	2.19E-5	1.382	0.0338	1.129	HMDB0243951	NA	Glycerolipids	down	up
M471T425_1	Glycyrrhetic acid (Enoxolone)	7.92E-4	1.872	0.0206	1.834	HMDB0011628	C02283	Prenol lipids	down	up
M317T281_A_1	Gossypetin	1.98E-5	1.998	0.0283	1.387	NA	C04109	NA	down	up
M315T538_1	Heneicosapentaenoic acid	8.97E-6	1.279	0.0139	1.154	NA	NA	NA	up	down
M268T331_1	Hepta-2,4-dienoylcarnitine	1.85E-4	2.116	0.03	1.824	HMDB0241690	NA	Fatty Acyls	down	up
M414T498_1	Heptadecanoyl carnitine	1.77E-6	1.503	0.0097	1.108	HMDB0006210	NA	Fatty Acyls	up	down

M249T516_C_1	Hexadecatrienoic acid	2.25E-6	1.757	0.0038	1.586	HMDB0302991	NA	Fatty Acyls	up	down
M260T259_1	Hexanoylcarnitine (C6:0)	1.85E-5	1.412	0.0047	1.439	HMDB0000756	NA	Fatty Acyls	up	down
M193T443_1	Hexylresorcinol	1.85E-6	1.577	0.0104	1.458	HMDB0032567	NA	Phenols	up	down
M261T236_1	Homovanillic acid sulfate	2.09E-6	2.126	0.0043	2.094	HMDB0011719	NA	Organic sulfuric acids and derivatives	down	up
M109T237_B_1	Hydroquinone	4.06E-4	1.969	0.0355	1.692	HMDB0002434	C00530	Phenols	down	up
M295T519_1	Hydroxyoctadeca-9,12-dienoic acid	2.51E-5	1.342	0.0262	1.135	NA	C08318	NA	up	down
M446T446_A_1	Icosa-5,8,10,12,14-pentaenoylcarnitine	7.52E-7	1.487	0.0039	1.301	HMDB0241594	NA	Fatty Acyls	up	down
M174T321_1	Indoleacetic acid	1.13E-7	1.383	0.0017	1.201	HMDB0000197	C00954	Indoles and derivatives	down	up
M186T344_B_1	Indoleacrylic acid	5.55E-4	1.815	0.0457	1.628	NA	NA	NA	down	up
M273T265_B_1	Isoferulic acid 3-sulfate	2.47E-6	2.022	5.71E-5	2.932	HMDB0041748	NA	Cinnamic acids and derivatives	down	up
M207T96_1	Kynurenine	3.34E-5	1.199	0.0024	1.444	HMDB0000684	C00328	Organooxygen compounds	up	down
M251T437_1	Linalyl hexanoate	2.12E-7	1.758	0.0074	1.693	HMDB0030429	NA	Prenol lipids	up	down

M279T466_1	Linoleate	3.66E-7	1.263	0.0074	1.266	HMDB0000673	C01595	Fatty Acyls	up	down
M424T467_A_1	Linoleylcarnitine (C18:2)	1.77E-7	1.445	0.002	1.391	HMDB0006461	NA	Fatty Acyls	up	down
M365T44_B_1	Maltose	1.50E-4	1.333	0.0018	2.080	HMDB0000055	C00185	Organooxygen compounds	down	up
M343T475_A_1	Medroxyprogesterone	5.33E-5	1.149	0.0074	1.196	HMDB0001939	C07119	Steroids and steroid derivatives	up	down
M343T475_B_1	Medrysone	5.33E-5	1.149	0.0074	1.196	HMDB0014398	C14643	Steroids and steroid derivatives	up	down
M365T44_C_1	Melibiose	1.50E-4	1.333	0.0018	2.080	HMDB0000048	C00252	Organooxygen compounds	down	up
M327T518_B_1	Methyl 8-[3-(2-hydroxyoctyl)oxiran-2-yl]octanoate	2.19E-5	1.382	0.0338	1.129	HMDB0246127	NA	Fatty Acyls	down	up
M209T490_1	Methyl dodecadienoate	0.0011	1.475	0.0031	1.568	HMDB0031014	NA	Fatty Acyls	up	down
M345T495_B_1	Methyl-[10]-shogaol	2.57E-5	1.448	0.0452	1.176	HMDB0031465	NA	Benzene and substituted derivatives	up	down
M317T281_B_1	Myricetin	1.98E-5	1.998	0.0283	1.387	HMDB0002755	C10107	Flavonoids	down	up
M227T543_1	Myristic acid	1.59E-4	1.170	0.004	1.411	HMDB0000806	C06424	Fatty Acyls	up	down
M312T92_1	N-(1-Deoxy-1-fructosyl)	2.72E-6	1.915	9.93E-4	2.180	HMDB0037841	NA	Carboxylic acids	down	up

	methionine							and derivatives		
M190T213_1	N-Acetylmethionine	5.11E-6	1.341	0.0169	1.152	HMDB0011745	C02712	Carboxylic acids and derivatives	down	up
M228T394_1	N-Decanoylglycine	4.03E-5	1.471	0.0036	1.641	HMDB0013267	NA	Carboxylic acids and derivatives	down	up
M198T351_1	N-Hexanoyl-homoserine lactone	4.91E-5	1.797	0.0409	1.546	NA	C21197	NA	up	down
M200T387_1	N-Octanoylglycine	2.10E-6	1.821	0.0499	1.548	HMDB0000832	NA	Carboxylic acids and derivatives	up	down
M372T449_2_B_1	N-Stearoyl Serine	2.17E-6	1.345	0.0179	1.318	HMDB0241949	NA	Carboxylic acids and derivatives	up	down
M446T446_B_1	N-oleoyl-tyrosine	7.52E-7	1.487	0.0039	1.301	HMDB0062337	NA	Carboxylic acids and derivatives	up	down
M344T413_B_1	N-palmitoyl-serine	2.05E-6	1.358	0.0075	1.399	HMDB0241932	NA	Carboxylic acids and derivatives	up	down
M420T432_1	N-palmitoyl-tyrosine	6.69E-4	1.749	0.0203	1.394	HMDB0062340	NA	Carboxylic acids and derivatives	up	down
M267T76_1	N5-(4-Methoxybenzyl)glutamine	6.78E-4	2.097	0.0491	1.833	HMDB0033598	NA	Carboxylic acids and derivatives	down	up
M183T385_A_1	Nepetalic acid	2.04E-6	1.931	0.0012	2.202	HMDB0036117	NA	Prenol lipids	down	up
M294T382_1	Nona-2,4,7-trienoylcarnitine	3.44E-5	1.504	0.0037	1.439	HMDB0241778	NA	Fatty Acyls	down	up
M344T413_A_1	O-lauroyl-carnitine	2.05E-6	1.358	0.0075	1.399	HMDB0002250	NA	Fatty Acyls	up	down

M372T449_2_A_1	O-myristoyl-carnitine	2.17E-6	1.345	0.0179	1.318	HMDB0005066	NA	Fatty Acyls	up	down
M426T488_1	O-oleoyl-carnitine	1.05E-6	1.450	0.0254	1.136	HMDB0005065	NA	Fatty Acyls	up	down
M309T484_D_1	Octadeca-2,4-dienedioic acid	1.05E-4	1.368	0.0406	1.085	HMDB0242188	NA	NA	down	up
M288T328_1	Octanoylcarnitine (C8:0)	1.16E-4	1.421	0.026	1.214	HMDB0000791	C02838	Fatty Acyls	up	down
M247T496_E_1	Octyl phenylacetate	1.67E-7	1.578	0.0019	1.516	HMDB0037713	NA	Fatty Acyls	up	down
M319T411_C_1	Oranabol	7.74E-4	1.222	0.0028	1.741	HMDB0006027	C14665	Steroids and steroid derivatives	up	down
M400T482_2_1	Palmitoylcarnitine (C16:0)	6.19E-7	1.169	0.0066	1.037	HMDB0000222	C02990	Fatty Acyls	up	down
M165T416_B_1	Pentyl 2-furyl ketone	2.02E-7	2.112	0.0105	1.370	HMDB0032462	NA	Organooxygen compounds	up	down
M193T413_A_1	Pentyl benzoate	1.05E-4	1.541	0.0125	1.419	HMDB0303724	NA	Benzene and substituted derivatives	down	up
M93T298_1	Phenol	3.56E-5	2.041	0.0237	1.574	HMDB0000228	C00146	Phenols	down	up
M433T340_A_1	Phlorizin chalcone	0.0013	2.142	0.0184	2.511	NA	C16406	NA	down	up
M153T307_A_1	Phloroglucinaldehyde	1.06E-4	2.063	0.02	1.828	HMDB0125090	NA	Phenols	down	up
M159T248_	Pimelic acid	2.64E-6	1.296	0.0039	1.229	HMDB0000857	C02656	Fatty Acyls	down	up

A_1										
M253T464_1	Prohydrojasmon	6.29E-5	1.959	0.0229	1.998	NA	C18538	NA	up	down
M109T237_A_1	Pyrocatechol	4.06E-4	1.969	0.0355	1.692	HMDB0000957	C00090	Phenols	down	up
M301T96_1	Pyrogallol-2-O-glucuronide	4.24E-5	2.008	7.88E-4	2.653	HMDB0060017	NA	Organooxygen compounds	down	up
M317T281_D_1	Quercetagetin	1.98E-5	1.998	0.0283	1.387	NA	C10122	NA	down	up
M447T310_A_1	Quercetin 7-rhamnoside	5.79E-4	2.075	0.0039	2.794	HMDB0257019	NA	Flavonoids	down	up
M137T299_1	Salicylic acid	6.04E-6	2.107	0.0103	1.630	HMDB0001895	C00805	Benzene and substituted derivatives	down	up
M275T518_A_1	Stearidonic acid	1.13E-4	1.193	0.0205	1.292	HMDB0006547	C16300	Fatty Acyls	up	down
M319T411_A_1	Steviol	7.74E-4	1.222	0.0028	1.741	HMDB0036706	C20212	Prenol lipids	up	down
M345T495_E_1	Taxa-4(20),11(12)-dien-5alpha-acetoxy-10beta-ol	2.57E-5	1.448	0.0452	1.176	NA	C11898	NA	up	down
M193T413_C_1	Tert-butyl 4-methylbenzoate	1.05E-4	1.541	0.0125	1.419	HMDB0258839	NA	Benzene and substituted derivatives	down	up
M239T462_1	Tetradecanedioic acid	1.01E-7	1.876	0.0327	1.489	HMDB0000872	NA	Fatty Acyls	up	down

M365T44_A_1	Trehalose	1.50E-4	1.333	0.0018	2.080	HMDB0000975	C01083	Organooxygen compounds	down	up
M301T400_A_1	Tributyrin	9.94E-6	1.862	0.017	1.597	HMDB0031094	C13870	Glycerolipids	down	up
M265T221_1	Val-Phe	7.28E-4	1.535	0.0015	2.473	HMDB0029134	NA	Carboxylic acids and derivatives	down	up
M265T312_1	[4]-Gingerol	4.02E-4	1.836	0.0076	2.012	HMDB0302125	NA	Phenols	down	up
M321T450_1	[8]-Gingerol	4.11E-8	2.261	0.0025	1.888	HMDB0033615	C17495	Phenols	down	up
M433T340_B_1	butein 4'-beta-glucoside	0.0013	2.142	0.0184	2.511	NA	NA	NA	down	up
M211T510_1	cis-11-Methyl-2-dodecenoic acid	1.68E-4	1.328	0.0123	1.276	NA	C18206	NA	up	down
M225T429_2_C_1	delta-Tetradecalactone	1.49E-6	1.622	0.0132	1.441	HMDB0038056	NA	Lactones	up	down
M301T473_A_1	ent-6,16-Kauradien-19-oic acid	5.83E-7	1.468	0.0437	1.248	HMDB0036697	NA	Prenol lipids	up	down
M277T92_1	gamma-Glutamylmethionine	0.0012	1.413	0.0434	1.168	HMDB0034367	NA	Carboxylic acids and derivatives	down	up
M249T516_A_1	hexadeca-7,10,13-trienoate	2.25E-6	1.757	0.0038	1.586	HMDB0302990	NA	Fatty Acyls	up	down
M247T496_D_1	hexadecatetra-4,7,10,13-enoate	1.67E-7	1.578	0.0019	1.516	HMDB0253125	NA	Fatty Acyls	up	down
M309T484_	hydroperoxyoctadeca-(9,1	1.05E-4	1.368	0.0406	1.085	HMDB0303985	NA	Fatty Acyls	down	up

B_1	1,14)-trienoate									
M343T475_C_1	methoxyprogesterone	5.33E-5	1.149	0.0074	1.196	HMDB0254554	NA	Steroids and steroid derivatives	up	down
M433T242_1	pinobanksin 7-O-glucoside	3.10E-5	2.181	0.0199	1.704	NA	NA	NA	down	up
M223T501_1	tetradeca-3,5-dienoate	6.03E-5	1.869	0.0108	1.641	NA	NA	NA	up	down
M163T295_B_1	trans-3-Hydroxycinnamic acid	0.0023	1.252	1.31E-4	2.932	HMDB0001713	C12621	Cinnamic acids and derivatives	down	up
M301T390_1	xi-7-Hydroxyhexadecanedioic acid	2.72E-5	1.721	0.0092	1.589	HMDB0037830	NA	Fatty Acyls	down	up

Supplementary Table S2. Colonic Content shared differential metabolites (*n* = 328) identified in both the LPS vs CON and MET vs LPS

comparisons

Feature ID	Name	LPS vs CON P	LPS vs CON VIP	MET vs LPS P	MET vs LPS VIP	HMDB	KEGG	Class	LPS vs CON	MET vs LPS
M457T468_B_1	(+-)-Ethyl 3-hydroxy-2-methylbutyrate	3.94E-4	1.421	0.0335	1.424	HMDB0032256	NA	Steroids and steroid derivatives	down	up
M677T51_1	(1,4)-beta-xylopentaose	0.0077	1.368	0.037	1.633	NA	NA	NA	down	up
M477T557_A_1	(1-{[1-carboxylato-3-(trimethylammonio)propan-2- yl]oxy}-1-oxoicosa-5,7,11,14-tetraen-9-yl)peroxy	0.0033	1.240	0.0229	1.458	HMDB0241798	NA	Fatty Acyls	down	up
M477T557_B_1	(20-{[1-Carboxylato-3-(trimethylammonio)propan-2- yl]oxy}-20-oxoicosa-6,8,12,15-tetraen-10-yl)peroxy	0.0033	1.240	0.0229	1.458	HMDB0241799	NA	Fatty Acyls	down	up
M411T399_A_1	(3beta,22E,24R)-3-Hydroxyergosta-5,8,22-trien-7-one	2.32E-4	1.207	0.0161	1.089	HMDB0032106	NA	Steroids and steroid derivatives	up	down
M195T394_A_1	(S,Z)-Lyratol acetate	1.43E-4	1.024	0.0188	1.096	HMDB0031840	NA	Carboxylic acids and derivatives	down	up
M231T320_	1,2-dibutyrin	0.0041	1.027	0.0173	1.388	HMDB0304007	NA	Glycerolipids	down	up

1										
M405T299_C_1	1-(2,3-Dihydroxyphenyl)-2-phenyl-2-[3,4,5-trihydroxy-6-(hydroxymethyl)oxan-2-yl]oxyethanone	5.13E-4	1.327	0.0023	2.033	HMDB0260230	NA	Stilbenes	down	up
M195T394_E_1	1-(2-Furanyl)-1-octanone	1.43E-4	1.024	0.0188	1.096	HMDB0040439	NA	Organooxygen compounds	down	up
M494T383_1	1-(9Z-Nonadecenoyl)-glycero-3-phosphoethanolamine	0.0058	1.182	0.0037	1.551	HMDB0243799	NA	Glycerophospholipids	up	down
M417T540_C_1	11'-Carboxy-alpha-chromanol	2.88E-4	1.329	7.99E-4	1.276	HMDB0012515	NA	Prenol lipids	up	down
M347T285_B_1	11-Deoxycortisol	3.56E-6	1.958	0.0125	1.144	HMDB0000015	C05488	Steroids and steroid derivatives	up	down
M347T285_D_1	11alpha,15alpha-Dihydroxypregn-4-ene-3,20-dione	3.56E-6	1.958	0.0125	1.144	NA	C15002	NA	up	down
M317T238_A_1	11alpha,17beta-Dihydroxy-17alpha-methylandrosta-1,4-dien-3-one	4.61E-4	1.050	0.0036	1.389	NA	C14887	NA	down	up
M349T287_A_1	11beta,21-Dihydroxy-5beta-pregnane-3,20-dione	8.34E-8	2.060	0.0225	1.246	HMDB0006757	C05475	Steroids and steroid derivatives	up	down
M349T341_A_1	12-Oxo-20-hydroxy-leukotriene B4	1.11E-7	1.441	0.0099	1.084	HMDB0012552	NA	Fatty Acyls	up	down
M365T309_B_1	12-oxo-10,11-dihydro-20-COOH-LTB4	1.54E-8	1.696	0.0074	1.157	HMDB0012549	NA	Fatty Acyls	up	down

M365T309_A_1	12-oxo-20-dihydroxy-leukotriene B4	1.54E-8	1.696	0.0074	1.157	HMDB0012551	NA	Fatty Acyls	up	down
M385T477_1	12alpha-Hydroxy-3-oxochola-4,6-dienoate	2.32E-5	2.036	5.13E-4	2.163	HMDB0000385	C15569	Steroids and steroid derivatives	up	down
M333T587_A_1	13,16,19-Docosatrienoic acid	1.41E-4	2.075	0.0062	1.364	HMDB0002823	C16534	Fatty Acyls	up	down
M349T341_B_1	15-dehydro-13,14-dihydro-prostaglandin E3	1.11E-7	1.441	0.0099	1.084	NA	NA	NA	up	down
M315T341_A_1	17-Ethynyl-10-hydroxy-19-nortestosterone	1.03E-7	1.473	0.0049	1.203	NA	C14670	NA	up	down
M349T287_B_1	17alpha,21-Dihydroxypregnenolone	8.34E-8	2.060	0.0225	1.246	HMDB0006762	C05487	Steroids and steroid derivatives	up	down
M317T238_B_1	17beta-Hydroxy-17-methylandrost-4-ene-3,11-dione	4.61E-4	1.050	0.0036	1.389	NA	C14615	NA	down	up
M317T348_B_1	17beta-Hydroxyestr-5(10)-en-3-one acetate	1.02E-7	1.474	0.0025	1.198	NA	C15282	NA	up	down
M347T285_C_1	19-Hydroxydeoxycorticosterone	3.56E-6	1.958	0.0125	1.144	HMDB0012612	NA	Steroids and steroid derivatives	up	down
M317T348_A_1	19-Nordeoxycorticosterone	1.02E-7	1.474	0.0025	1.198	HMDB0244837	NA	Steroids and steroid derivatives	up	down
M285T308_B_1	2',4'-dimethoxy 4-hydroxy chalcone	3.68E-4	1.001	0.0026	1.078	NA	NA	NA	up	down
M209T298_A_1	2'-Hydroxy-4',6'-dimethoxy-3'-methylacetophenone	8.90E-5	1.099	0.0219	1.057	HMDB0034047	NA	Organooxygen compounds	down	up
M285T308_	2'-Hydroxy-4',6'-dimethox	3.68E-4	1.001	0.0026	1.078	HMDB0033691	NA	Linear	up	down

D_1	ychalcone							1,3-diarylpropanoids		
M403T290_D_1	2'-hydroxy 3,6,7,3',4'-pentamethylquercetagetin	7.25E-5	1.459	0.0065	1.604	NA	NA	NA	down	up
M139T221_E_1	2,3,5-Trihydroxytoluene	8.24E-7	1.067	2.46E-4	1.205	NA	C03338	NA	up	down
M225T236_D_1	2,4,6-Trimethoxyphenyl acetate	2.89E-7	1.325	0.0126	1.072	HMDB0041560	NA	Phenol esters	down	up
M153T321_A_1	2,4,6-trihydroxybenzaldehyde	0.0038	1.444	0.0272	1.714	HMDB0125090	NA	Phenols	down	up
M153T321_B_1	2,6-Dihydroxybenzoate	0.0038	1.444	0.0272	1.714	HMDB0013676	C21298	Benzene and substituted derivatives	down	up
M188T75_C_1	2-Amino-6-oxoheptanedioate	0.0025	1.045	0.0358	1.367	NA	C03871	NA	up	down
M102T44_A_1	2-Aminobutyric acid	1.34E-4	1.341	0.0046	1.707	HMDB0000452	C02261	Carboxylic acids and derivatives	up	down
M102T44_B_1	2-Aminoisobutyric acid	1.34E-4	1.341	0.0046	1.707	HMDB0001906	C03665	Carboxylic acids and derivatives	up	down
M131T262_C_1	2-Hydroxy-3-methylpentanoic acid	0.0012	1.496	0.0493	1.708	HMDB0000317	NA	Fatty Acyls	down	up
M145T330_A_1	2-Hydroxyheptanoic acid	8.45E-4	1.588	0.0426	1.574	NA	NA	NA	down	up
M131T262_	2-Hydroxyhexanoic acid	0.0012	1.496	0.0493	1.708	HMDB0001624	NA	Fatty Acyls	down	up

A_1										
M159T356_A_1	2-Hydroxyoctanoic acid	8.05E-4	1.143	2.41E-4	2.124	HMDB0000711	NA	Fatty Acyls	down	up
M139T221_C_1	2-Methoxy-1,3-benzenediol	8.24E-7	1.067	2.46E-4	1.205	HMDB0133970	NA	Phenols	up	down
M209T298_E_1	2-Methoxy-3-(4-methoxyphenyl)propanoic acid	8.90E-5	1.099	0.0219	1.057	HMDB0039428	NA	Phenylpropanoic acids	down	up
M139T221_B_1	2-methoxyhydroquinone	8.24E-7	1.067	2.46E-4	1.205	NA	NA	NA	up	down
M347T285_A_1	21-Deoxycortisol	3.56E-6	1.958	0.0125	1.144	HMDB0004030	C05497	Steroids and steroid derivatives	up	down
M383T507_1	25-Hydroxyvitamin D3	1.64E-4	1.037	0.0303	1.001	HMDB0003550	C01561	Steroids and steroid derivatives	down	up
M912T463_D_1	28-[Glucosyl-(1->6)-glucosyl]oleanolic acid 3-arabinoside	0.0016	1.694	0.036	1.875	HMDB0036362	NA	Prenol lipids	down	up
M387T317_E_1	3'-Hydroxy-4',5,6,7,8-pentamethoxyflavone	0.002	1.422	0.022	1.615	HMDB0029545	NA	Flavonoids	down	up
M427T508_1	3,24-dioxo-cholest-4-en-26-oate	1.69E-6	2.024	0.0025	2.415	NA	NA	NA	up	down
M153T321_C_1	3,4,5-Trihydroxybenzaldehyde	0.0038	1.444	0.0272	1.714	HMDB0246043	NA	Phenols	down	up
M153T206_A_1	3,4-Dihydroxybenzoic acid (Protocatechuic acid)	0.034	1.324	0.0284	1.788	HMDB0001856	C00230	Benzene and substituted derivatives	down	up

M181T254_1	3,4-Dihydroxyhydrocinnamic acid	7.09E-4	1.308	0.0455	1.258	HMDB0000423	C10447	Phenylpropanoic acids	down	up
M209T298_C_1	3,4-Dihydroxyphenylvaleric acid	8.90E-5	1.099	0.0219	1.057	HMDB0029233	NA	Phenols	down	up
M407T594_1	3,4-Dimethyl-5-pentyl-2-furanpentadecanoic acid	9.11E-6	1.229	0.0107	1.136	HMDB0112081	NA	Fatty Acyls	up	down
M385T282_1	3,5-Dihydroxyphenylvaleric acid glucuronide	8.80E-6	1.892	0.0073	1.693	HMDB0240451	NA	Saccharolipids	up	down
M403T290_E_1	3,6,7,2',4'-pentamethylquercetagetin	7.25E-5	1.459	0.0065	1.604	NA	NA	NA	down	up
M387T317_B_1	3,6,7,3',4'-pentamethylquercetagetin	0.002	1.422	0.022	1.615	HMDB0030095	NA	Flavonoids	down	up
M387T317_C_1	3,7,3',4',5'-pentamethylmyricetin	0.002	1.422	0.022	1.615	NA	NA	NA	down	up
M165T297_3_A_1	3-(2-Hydroxyphenyl)propanoic acid	0.0042	2.020	0.0134	2.636	HMDB0033752	C01198	Phenylpropanoic acids	down	up
M165T297_3_B_1	3-(3-Hydroxyphenyl)propanoic acid	0.0042	2.020	0.0134	2.636	HMDB0000375	C11457	Phenylpropanoic acids	down	up
M225T236_A_1	3-(4-Hydroxy-3-methoxyphenyl)-2-methyllactic acid	2.89E-7	1.325	0.0126	1.072	HMDB0060736	NA	Phenylpropanoic acids	down	up
M148T81_C_1	3-Aminopentanedioate	1.18E-5	1.437	0.0105	1.509	NA	C05574	NA	up	down
M239T272_1	3-CMPFP	3.13E-5	1.352	1.94E-4	1.451	HMDB0061643	NA	Fatty Acyls	down	up
M137T256_	3-Hydroxybenzoic acid	1.63E-4	1.447	2.83E-4	2.178	HMDB0002466	C00587	Benzene and	down	up

A_1								substituted derivatives		
M311T303_C_1	3-Hydroxychavicol 1-glucoside	3.28E-4	1.186	2.10E-4	2.189	HMDB0034861	NA	Organooxygen compounds	up	down
M159T356_B_1	3-Hydroxyoctanoic acid	8.05E-4	1.143	2.41E-4	2.124	HMDB0001954	C20793	Hydroxy acids and derivatives	down	up
M159T255_B_1	3-Methyladipic acid	1.81E-6	1.070	0.0057	1.103	HMDB0000555	NA	Fatty Acyls	down	up
M912T463_A_1	3-O-{beta-Glucopyranosyl(1_2)-[alpha-arabinopyranosyl(1_6)]-beta-glucopyranosyl}-oleanolic acid	0.0016	1.694	0.036	1.875	HMDB0304603	NA	Prenol lipids	down	up
M94T71_1	3-Pyridol	2.31E-4	1.365	5.96E-4	2.113	NA	NA	NA	up	down
M243T333_B_1	3-hydroxy-2-oxopropyl 7-methyl-3-oxooctanoate	7.11E-4	1.204	0.002	1.521	NA	NA	NA	down	up
M349T287_D_1	3alpha,17alpha-Dihydroxy-5beta-pregnane-11,20-dione	8.34E-8	2.060	0.0225	1.246	NA	C14598	NA	up	down
M349T287_C_1	3alpha,21-Dihydroxy-5beta-pregnane-11,20-dione	8.34E-8	2.060	0.0225	1.246	HMDB0006755	C05478	Steroids and steroid derivatives	up	down
M417T548_C_1	3alpha,7alpha-Dihydroxy-5beta-cholestan-26-al	6.70E-8	1.455	0.0023	1.139	HMDB0006894	C05445	Steroids and steroid derivatives	up	down
M455T461_1	3beta-3-Hydroxy-11-oxolanosta-8,24-dien-26-al	1.63E-4	1.214	0.0132	1.371	HMDB0035406	NA	Prenol lipids	down	up
M341T331_	4',5,6,7-Tetramethoxyflav	0.002	1.177	0.0335	1.292	HMDB0030575	C14472	Flavonoids	down	up

2_D_1	one									
M341T331_2_C_1	4,2'-dihydroxy-3,3'-dimethoxy-5'-(2''-carboxyvinyl) stilbene	0.002	1.177	0.0335	1.292	NA	NA	NA	down	up
M379T603_1	4,9,13,17,21-pentamethyldocosa-2,4,6,8,10,12,16,20-octaenal	1.17E-5	1.410	0.0051	1.411	NA	NA	NA	up	down
M194T297_2_D_1	4-(Butylamino)benzoic acid	4.19E-7	1.737	3.40E-4	1.851	HMDB0246301	NA	Benzene and substituted derivatives	up	down
M102T44_D_1	4-Aminobutyric acid (GABA)	1.34E-4	1.341	0.0046	1.707	HMDB0000112	C00334	Carboxylic acids and derivatives	up	down
M165T242_1	4-Coumarate	7.95E-4	1.486	0.0089	2.123	HMDB0002035	C00811	Cinnamic acids and derivatives	down	up
M195T394_D_1	4-Hexyloxyphenol	1.43E-4	1.024	0.0188	1.096	NA	C14305	NA	down	up
M225T236_B_1	4-Hydroxy-(3',4'-dihydroxyphenyl)-valeric acid	2.89E-7	1.325	0.0126	1.072	HMDB0041679	NA	Hydroxy acids and derivatives	down	up
M225T236_E_1	4-Hydroxy-5-(3',5'-dihydroxyphenyl)-valeric acid	2.89E-7	1.325	0.0126	1.072	HMDB0240455	NA	Hydroxy acids and derivatives	down	up
M401T224_1	4-Hydroxy-5-(3',5'-dihydroxyphenyl)-valeric acid glucuronide	1.32E-5	1.211	0.0169	1.054	HMDB0240456	NA	Organooxygen compounds	up	down
M137T256_C_1	4-Hydroxybenzoic acid	1.63E-4	1.447	2.83E-4	2.178	HMDB0000500	C00156	Benzene and substituted	down	up

								derivatives		
M311T303_B_1	4-Hydroxycinnamyl alcohol 4-glucoside	3.28E-4	1.186	2.10E-4	2.189	NA	C05855	NA	up	down
M167T71_2_1	4-Hydroxyphenylglycolic acid	2.46E-5	1.148	7.04E-4	1.511	HMDB0000822	C03198	Phenols	down	up
M315T341_D_1	4-Oxo-13-cis-retinoate	1.03E-7	1.473	0.0049	1.203	HMDB0012789	NA	Prenol lipids	up	down
M411T634_A_1	4alpha-Methyl-5alpha-ergosta-8,14,24(28)-trien-3beta-ol	5.38E-5	1.101	0.0228	1.001	HMDB0006928	C11508	Steroids and steroid derivatives	down	up
M411T634_B_1	4alpha-methyl ergosta-8,14,25(27)-trienol	5.38E-5	1.101	0.0228	1.001	NA	NA	NA	down	up
M387T317_A_1	5'-Hydroxyaverantin	0.002	1.422	0.022	1.615	NA	C20500	NA	down	up
M385T318_D_1	5'-Oxoaverantin	2.38E-4	1.416	0.0062	1.471	NA	C20502	NA	down	up
M341T3312_B_1	5,6,7,4'-Tetramethoxyisoflavone	0.002	1.177	0.0335	1.292	NA	C15076	NA	down	up
M281T316_E_1	5,7-Dimethoxyflavone	5.95E-4	1.502	0.0038	2.141	HMDB0036620	C10029	Flavonoids	down	up
M385T295_D_1	5-8'-Dehydrodiferulic acid	4.23E-4	1.515	0.0099	1.719	HMDB0301719	NA	Stilbenes	down	up
M211T252_1	5-Carboxyvanillic acid	0.0024	1.496	0.0082	1.896	NA	C18338	NA	down	up

M387T317_D_1	5-Hydroxy-3,3',4',7,8-pentamethoxyflavone	0.002	1.422	0.022	1.615	HMDB0029227	NA	Flavonoids	down	up
M148T81_D_1	5-Hydroxy-4-oxo-norvaline	1.18E-5	1.437	0.0105	1.509	HMDB0246802	NA	Carboxylic acids and derivatives	up	down
M281T316_D_1	5-Hydroxy-7-methoxy-6-methylflavone	5.95E-4	1.502	0.0038	2.141	HMDB0037255	NA	Flavonoids	down	up
M313T309_B_1	5-Methoxy-7-(4-hydroxyphenyl)-1-phenyl-3-heptanone	4.34E-5	1.295	0.0074	1.245	HMDB0033294	NA	Diarylheptanoids	up	down
M174T51_3_1	5-[(1-Iminoethyl)amino]-2-aminopentanoic acid	0.0098	1.082	0.0118	2.121	HMDB0246210	NA	Carboxylic acids and derivatives	up	down
M579T291_1	6"-p-Coumaroylprunin	1.56E-4	1.030	0.0049	1.018	HMDB0037583	NA	Flavonoids	up	down
M140T71_1	6-Hydroxynicotinic acid	2.92E-4	1.592	0.0032	2.154	HMDB0002658	C01020	Pyridines and derivatives	up	down
M287T305_2_1	6-Hydroxypentadecanedioic acid	0.0026	1.085	2.68E-4	1.816	HMDB0031885	NA	Fatty Acyls	up	down
M347T285_E_1	6beta,11alpha-Dihydroxyprogesterone	3.56E-6	1.958	0.0125	1.144	NA	C15363	NA	up	down
M281T316_C_1	7,8-dimethoxy-flavone	5.95E-4	1.502	0.0038	2.141	NA	NA	NA	down	up
M401T347_B_1	7-Ketocholesterol	0.0041	1.248	0.0111	1.717	HMDB0000501	NA	Steroids and steroid derivatives	down	up
M417T548_B_1	7alpha,12alpha-Dihydroxy-5beta-cholestan-3-one	6.70E-8	1.455	0.0023	1.139	HMDB0006886	C05453	Steroids and steroid derivatives	up	down

M417T548_D_1	7alpha,24-dihydroxy-5beta-cholestan-3-one	6.70E-8	1.455	0.0023	1.139	HMDB0062419	NA	Steroids and steroid derivatives	up	down
M417T548_E_1	7alpha,26-dihydroxy-5beta-cholestan-3-one	6.70E-8	1.455	0.0023	1.139	HMDB0062420	NA	Steroids and steroid derivatives	up	down
M385T318_C_1	8-8'-Dehydrodiferulic acid	2.38E-4	1.416	0.0062	1.471	HMDB0029277	NA	NA	down	up
M331T216_1	8-Amino-7-oxononanoylcarnitine	2.55E-4	1.987	4.70E-4	3.262	HMDB0241866	NA	Fatty Acyls	down	up
M329T395_1	9,12,13-TriHOME	1.10E-4	1.461	0.0017	1.833	HMDB0004708	C14833	Fatty Acyls	down	up
M305T367_1	9-Chloro-10-hydroxyhexadecanoic acid	1.97E-6	1.595	0.0163	1.366	NA	C13949	NA	up	down
M243T333_A_1	9-hydroperoxytraumatin	7.11E-4	1.204	0.002	1.521	NA	NA	NA	down	up
M304T250_1	Acylcarnitine C8:0,OH	0.003	1.420	0.007	3.350	NA	NA	NA	up	down
M912T463_C_1	Akeboside Stf	0.0016	1.694	0.036	1.875	NA	C17546	NA	down	up
M395T237_C_1	Aklanonate	2.79E-4	1.413	7.53E-4	1.870	NA	C12421	NA	up	down
M401T347_A_1	Alfacalcidol	0.0041	1.248	0.0111	1.717	HMDB0015504	NA	Steroids and steroid derivatives	down	up
M366T433_1	Alpha-Linoleoylcholine [M]	7.10E-6	1.926	0.012	1.611	HMDB0013213	NA	Organonitrogen compounds	down	up
M365T364_	Arbaprostil	1.02E-6	1.842	0.0041	1.521	HMDB0248565	NA	Fatty Acyls	up	down

3_A_1										
M942T461_A_1	Arvensoside A	5.42E-4	1.677	0.0115	2.023	NA	C08923	NA	down	up
M942T461_C_1	Arvensoside D	5.42E-4	1.677	0.0115	2.023	HMDB0039406	NA	Prenol lipids	down	up
M605T576_A_1	Asiminenin A	5.82E-5	2.252	0.0262	2.052	HMDB0029959	NA	Fatty Acyls	down	up
M399T254_1	Atractyloside C	2.14E-5	1.447	0.0393	1.126	NA	C17860	NA	up	down
M395T237_B_1	Auraviketone	2.79E-4	1.413	7.53E-4	1.870	NA	C12417	NA	up	down
M209T298_B_1	Bancroftinone	8.90E-5	1.099	0.0219	1.057	HMDB0041406	NA	Organooxygen compounds	down	up
M405T299_A_1	Benzyl 2,6-dihydroxybenzoate 2-glucoside	5.13E-4	1.327	0.0023	2.033	HMDB0038330	NA	Organooxygen compounds	down	up
M194T297_2_C_1	Butamben	4.19E-7	1.737	3.40E-4	1.851	HMDB0249473	C07875	Benzene and substituted derivatives	up	down
M145T330_B_1	Butyllactate	8.45E-4	1.588	0.0426	1.574	HMDB0040254	NA	Carboxylic acids and derivatives	down	up
M355T226_B_1	Caffeic acid 3-O-glucuronide	0.0039	1.441	0.0481	1.004	HMDB0041705	NA	Organooxygen compounds	up	down
M355T226_C_1	Caffeic acid 4-O-glucuronide	0.0039	1.441	0.0481	1.004	HMDB0041707	NA	Isoflavonoids	up	down

M355T226_A_1	Caffeoyl C1-glucuronide	0.0039	1.441	0.0481	1.004	HMDB0041709	NA	Cinnamic acids and derivatives	up	down
M942T461_D_1	Calenduloside B	5.42E-4	1.677	0.0115	2.023	HMDB0039413	NA	Prenol lipids	down	up
M283T337_A_1	Calycosin	0.0031	1.550	0.016	1.993	HMDB0249566	C01562	Isoflavonoids	down	up
M403T290_C_1	Cassiaside	7.25E-5	1.459	0.0065	1.604	HMDB0033913	NA	Naphthopyrans	down	up
M417T548_A_1	Cholest-5-ene-3beta,7alpha,24-triol	6.70E-8	1.455	0.0023	1.139	HMDB0011644	C15518	Steroids and steroid derivatives	up	down
M385T318_A_1	Cleomiscosin A	2.38E-4	1.416	0.0062	1.471	NA	C09922	NA	down	up
M661T401_B_1	Coproporphyrinogen I	1.76E-4	1.297	0.0309	1.247	HMDB0002158	C05768	Tetrapyrroles and derivatives	up	down
M661T401_A_1	Coproporphyrinogen III	1.76E-4	1.297	0.0309	1.247	HMDB0001261	C03263	Tetrapyrroles and derivatives	up	down
M329T328_1	Corticosterone	1.58E-7	2.138	0.0057	1.340	HMDB0001547	C02140	Steroids and steroid derivatives	up	down
M594T598_1	DG(16:0/18:2(9Z,12Z)/0:0)	1.11E-4	1.577	0.0053	1.746	HMDB0007103	NA	Fatty Acyls	down	up
M385T318_B_1	Daphneticin	2.38E-4	1.416	0.0062	1.471	NA	C09924	NA	down	up
M385T295_B_1	Dehydrodiferulic dilactone	4.23E-4	1.515	0.0099	1.719	HMDB0033876	NA	Lignan lactones	down	up
M349T287_	Deoxycortisol	8.34E-8	2.060	0.0225	1.246	HMDB0251005	NA	Steroids and	up	down

E_1								steroid derivatives		
M165T283_1	Desaminotyrosine	3.96E-4	1.569	0.0041	1.997	HMDB0002199	C01744	Phenylpropanoic acids	down	up
M331T364_1	Desoxycortone	3.05E-7	1.764	0.0029	1.445	HMDB0000016	C03205	Steroids and steroid derivatives	up	down
M89T50_A_1	Dihydroxyacetone	4.11E-4	1.047	8.97E-4	1.505	HMDB0001882	C00184	Organooxygen compounds	down	up
M417T540_B_1	Diisononyl phthalate	2.88E-4	1.329	7.99E-4	1.276	HMDB0251352	C15221	Benzene and substituted derivatives	up	down
M102T44_C_1	Dimethylglycine	1.34E-4	1.341	0.0046	1.707	HMDB0000092	C01026	Carboxylic acids and derivatives	up	down
M417T540_D_1	Dinonyl phthalate	2.88E-4	1.329	7.99E-4	1.276	HMDB0251424	NA	Benzene and substituted derivatives	up	down
M333T587_B_1	Docosatrienoic acid	1.41E-4	2.075	0.0062	1.364	HMDB0031099	C21940	Fatty Acyls	up	down
M303T355_1	Dolichol phosphate	9.40E-7	1.583	0.0152	1.287	HMDB0006353	NA	Prenol lipids	up	down
M380T267_1	Donepezil	5.88E-6	1.667	0.0044	1.612	HMDB0005041	NA	Piperidines	down	up
M405T299_B_1	Edulisin VI	5.13E-4	1.327	0.0023	2.033	HMDB0041265	NA	Coumarins and derivatives	down	up
M325T284_1	Escitalopram (Drug)	1.03E-5	1.384	0.01	1.311	HMDB0005028	C07572	Benzene and substituted	up	down

								derivatives		
M287T419_1	Ethylsuberenol	9.75E-5	1.583	1.10E-4	2.671	HMDB0039044	NA	Coumarins and derivatives	up	down
M245T290_A_1	ExMrn00005863	0.0068	1.625	0.0225	2.242	NA	NA	NA	down	up
M611T372_A_1	ExMrn00006313	7.00E-7	1.453	0.0278	1.140	NA	NA	NA	up	down
M611T372_B_1	ExMrn00006314	7.00E-7	1.453	0.0278	1.140	NA	NA	NA	up	down
M609T383_A_1	ExMrn00006324	1.09E-5	1.579	0.0375	1.221	NA	NA	NA	up	down
M609T383_C_1	ExMrn00006325	1.09E-5	1.579	0.0375	1.221	NA	NA	NA	up	down
M609T383_D_1	ExMrn00006328	1.09E-5	1.579	0.0375	1.221	NA	NA	NA	up	down
M609T366_A_1	ExMrn00006329	5.28E-7	1.558	0.0156	1.076	NA	NA	NA	up	down
M609T366_B_1	ExMrn00006330	5.28E-7	1.558	0.0156	1.076	NA	NA	NA	up	down
M609T366_C_1	ExMrn00006331	5.28E-7	1.558	0.0156	1.076	NA	NA	NA	up	down
M609T383_B_1	ExMrn00006337	1.09E-5	1.579	0.0375	1.221	NA	NA	NA	up	down
M609T366_D_1	ExMrn00006343	5.28E-7	1.558	0.0156	1.076	NA	NA	NA	up	down

M609T366_E_1	ExMrn00006344	5.28E-7	1.558	0.0156	1.076	NA	NA	NA	up	down
M503T422_E_1	ExMrn00010898	0.0222	1.050	0.0072	1.841	NA	NA	NA	up	up
M325T215_1	ExMrn00011220	0.0109	1.054	0.0099	2.587	NA	NA	NA	up	down
M503T422_A_1	ExMrn00030183	0.0222	1.050	0.0072	1.841	NA	NA	NA	up	up
M503T422_B_1	ExMrn00030184	0.0222	1.050	0.0072	1.841	NA	NA	NA	up	up
M503T422_C_1	ExMrn00030185	0.0222	1.050	0.0072	1.841	NA	NA	NA	up	up
M320T324_2_C_1	ExMrn00048764 [M]	1.28E-4	1.262	0.0067	1.263	NA	NA	NA	up	down
M320T324_2_A_1	ExMrn00048767 [M]	1.28E-4	1.262	0.0067	1.263	NA	NA	NA	up	down
M320T324_2_D_1	ExMrn00048768 [M]	1.28E-4	1.262	0.0067	1.263	NA	NA	NA	up	down
M320T324_2_B_1	ExMrn00048773 [M]	1.28E-4	1.262	0.0067	1.263	NA	NA	NA	up	down
M320T324_2_E_1	ExMrn00048774 [M]	1.28E-4	1.262	0.0067	1.263	NA	NA	NA	up	down
M597T420_1	ExMrn00063519	7.28E-4	1.103	0.0297	1.008	NA	NA	NA	up	down
M503T422_	ExMrn00063739	0.0222	1.050	0.0072	1.841	NA	NA	NA	up	up

D_1										
M245T290_B_1	ExMrn00068773	0.0068	1.625	0.0225	2.242	NA	NA	NA	down	up
M413T335_B_1	ExMrn00076430	1.66E-7	1.447	0.0105	1.040	NA	NA	NA	up	down
M413T335_C_1	ExMrn00076434	1.66E-7	1.447	0.0105	1.040	NA	NA	NA	up	down
M413T335_E_1	ExMrn00076438	1.66E-7	1.447	0.0105	1.040	NA	NA	NA	up	down
M413T335_A_1	ExMrn00076780	1.66E-7	1.447	0.0105	1.040	NA	NA	NA	up	down
M413T335_D_1	ExMrn00076783	1.66E-7	1.447	0.0105	1.040	NA	NA	NA	up	down
M475T322_1	ExMrn00081097	0.0014	1.497	0.0279	1.753	NA	NA	NA	down	up
M377T323_1	ExMrn00086956	2.15E-6	1.551	0.0058	1.436	NA	NA	NA	up	up
M627T329_1	ExMrn00092029	7.17E-4	1.555	0.0144	1.181	NA	NA	NA	up	down
M417T532_A_1	ExMrn00110279	5.75E-6	1.213	0.0037	1.238	NA	NA	NA	up	down
M417T532_B_1	ExMrn00110280	5.75E-6	1.213	0.0037	1.238	NA	NA	NA	up	down
M475T445_A_1	ExMrn00120789	3.70E-4	1.192	0.0343	1.062	NA	NA	NA	up	down

M475T445_D_1	ExMrn00120798	3.70E-4	1.192	0.0343	1.062	NA	NA	NA	up	down
M475T445_B_1	ExMrn00120799	3.70E-4	1.192	0.0343	1.062	NA	NA	NA	up	down
M475T445_E_1	ExMrn00120800	3.70E-4	1.192	0.0343	1.062	NA	NA	NA	up	down
M475T445_C_1	ExMrn00120801	3.70E-4	1.192	0.0343	1.062	NA	NA	NA	up	down
M193T296_2_1	Ferulic acid	5.39E-4	1.203	0.0136	1.351	HMDB0000954	C01494	Cinnamic acids and derivatives	down	up
M405T299_D_1	Flacourtin	5.13E-4	1.327	0.0023	2.033	HMDB0039172	NA	Organooxygen compounds	down	up
M784T264_1	Flavin adenine dinucleotide (FAD)	0.0017	1.123	0.0443	1.218	HMDB0001248	C00016	Flavin nucleotides	up	down
M139T221_D_1	Furfuryl acetate	8.24E-7	1.067	2.46E-4	1.205	HMDB0034246	NA	Heteroaromatic compounds	up	down
M455T416_1	Ganoderal B	1.58E-4	1.196	0.0302	1.221	HMDB0036002	NA	Prenol lipids	down	up
M555T560_A_1	Ganoderic acid Me	1.32E-6	1.885	0.018	1.239	HMDB0035333	NA	Prenol lipids	up	down
M555T560_B_1	Ganoderic acid R	1.32E-6	1.885	0.018	1.239	HMDB0035621	NA	Prenol lipids	up	down
M455T488_E_1	Ganoderic acid Y	0.0019	1.375	0.0083	1.765	HMDB0035090	NA	Prenol lipids	down	up
M455T488_	Ganoderiol F	0.0019	1.375	0.0083	1.765	HMDB0038707	NA	Prenol lipids	down	up

B_1										
M457T468_C_1	Ganodermanondiol	3.94E-4	1.421	0.0335	1.424	HMDB0033020	NA	Prenol lipids	down	up
M441T507_2_C_1	Ganoderol B	9.66E-4	1.199	0.0316	1.221	HMDB0035314	NA	Prenol lipids	down	up
M283T337_C_1	Genkwanin	0.0031	1.550	0.016	1.993	HMDB0252684	C10046	Flavonoids	down	up
M137T256_B_1	Gentisaldehyde	1.63E-4	1.447	2.83E-4	2.178	HMDB0004062	C05585	Organooxygen compounds	down	up
M153T206_B_1	Gentisic acid	0.034	1.324	0.0284	1.788	HMDB0000152	C00628	Benzene and substituted derivatives	down	up
M139T221_A_1	Gentisyl alcohol	8.24E-7	1.067	2.46E-4	1.205	NA	C10792	NA	up	down
M188T75_A_1	Glutarylglycine	0.0025	1.045	0.0358	1.367	HMDB0000590	NA	Carboxylic acids and derivatives	up	down
M89T50_B_1	Glyceraldehyde	4.11E-4	1.047	8.97E-4	1.505	HMDB0001051	C00577	Organooxygen compounds	down	up
M147T97_D_1	Glycerol 1-propanoate	3.85E-5	1.175	4.46E-4	1.602	HMDB0031639	NA	Glycerolipids	down	up
M283T337_B_1	Glycitein	0.0031	1.550	0.016	1.993	HMDB0005781	C14536	Isoflavonoids	down	up
M301T335_B_1	Hesperetin	0.0011	1.305	0.045	1.447	HMDB0005782	C01709	Flavonoids	up	down
M417T540_	Hexadecyl ferulate	2.88E-4	1.329	7.99E-4	1.276	HMDB0039317	NA	Cinnamic acids	up	down

A_1								and derivatives		
M195T394_C_1	Hexylresorcinol	1.43E-4	1.024	0.0188	1.096	HMDB0032567	NA	Phenols	down	up
M912T463_E_1	Hoduloside III	0.0016	1.694	0.036	1.875	HMDB0039072	NA	Fatty Acyls	down	up
M301T335_A_1	Homoeriodictyol	0.0011	1.305	0.045	1.447	HMDB0253205	C09756	Flavonoids	up	down
M181T243_1	Homovanillic acid	3.60E-4	1.822	0.0023	2.667	HMDB0000118	C05582	Phenols	down	up
M131T262_B_1	Hydroxyisocaproic acid	0.0012	1.496	0.0493	1.708	HMDB0000624	C03264	Fatty Acyls	down	up
M593T391_1	I-Urobilinogen	1.76E-7	1.669	0.0115	1.409	HMDB0001898	C05790	Tetrapyrroles and derivatives	up	down
M146T274_1	Indole-3-carbinol	0.0012	1.380	0.0275	1.386	HMDB0005785	NA	Indoles and derivatives	down	up
M174T324_1	Indoleacetic acid	0.0019	1.261	0.0256	1.386	HMDB0000197	C00954	Indoles and derivatives	down	up
M385T318_E_1	Irisflorentin	2.38E-4	1.416	0.0062	1.471	NA	C17958	NA	down	up
M194T297_2_B_1	Isobutamben	4.19E-7	1.737	3.40E-4	1.851	HMDB0253607	NA	Benzene and substituted derivatives	up	down
M324T320_B_1	Jasmonoyl-isoleucine	0.0013	1.630	0.0299	1.796	HMDB0029391	C18699	Carboxylic acids and derivatives	down	up
M795T472_	Jujubasaponin VI	0.0017	1.763	0.0061	2.329	HMDB0040946	NA	Prenol lipids	down	up

D_1										
M445T279_B_1	Kaempferide 3-rhamnoside	0.0048	1.331	0.0245	1.428	HMDB0040803	NA	Flavonoids	down	up
M441T507_2_A_1	Karounidiol	9.66E-4	1.199	0.0316	1.221	NA	C17394	NA	down	up
M942T461_E_1	Lemmatoxin	5.42E-4	1.677	0.0115	2.023	NA	C08958	NA	down	up
M279T557_3_1	Linoleic acid	8.30E-5	1.165	0.0471	1.105	HMDB0000673	C01595	Fatty Acyls	down	up
M455T488_C_1	Lucidal	0.0019	1.375	0.0083	1.765	HMDB0040454	NA	Benzene and substituted derivatives	down	up
M795T472_C_1	Mabiogenin 3-[rhamnosyl-(1->6)-glucoside]	0.0017	1.763	0.0061	2.329	HMDB0040943	NA	Steroids and steroid derivatives	down	up
M455T488_A_1	Mangiferonic acid	0.0019	1.375	0.0083	1.765	HMDB0035615	NA	Steroids and steroid derivatives	down	up
M455T488_D_1	Masticadienonic acid	0.0019	1.375	0.0083	1.765	HMDB0040508	NA	Prenol lipids	down	up
M194T297_2_A_1	Methoxyphedrine	4.19E-7	1.737	3.40E-4	1.851	HMDB0243795	NA	Organooxygen compounds	up	down
M389T538_1	Methyl 2-(10-heptadecenyl)-6-hydroxybenzoate	5.88E-6	1.275	0.0022	1.053	HMDB0038523	NA	Benzene and substituted derivatives	up	down
M315T341_	Methyl cis-p-coumarate	1.03E-7	1.473	0.0049	1.203	HMDB0041152	NA	Cinnamic acids	up	down

B_1	3-(3,7-dimethyl-2,6-octadienyl)							and derivatives		
M395T237_A_1	Methyl nogalonate	2.79E-4	1.413	7.53E-4	1.870	NA	C12415	NA	up	down
M385T252_1	Methylsyringin	6.48E-4	2.128	0.0351	2.056	HMDB0039570	NA	Organooxygen compounds	up	down
M147T97_C_1	Mevalonate	3.85E-5	1.175	4.46E-4	1.602	HMDB0000227	C00418	Fatty Acyls	down	up
M423T350_1	Monoketocholic acid	1.62E-4	1.087	0.0137	1.752	HMDB0254862	NA	Steroids and steroid derivatives	up	up
M311T303_A_1	Moringyne	3.28E-4	1.186	2.10E-4	2.189	HMDB0031724	NA	Organooxygen compounds	up	down
M299T502_1	Myrigalon B	0.0023	1.253	0.0037	1.914	HMDB0037246	NA	Linear 1,3-diarylpropanoids	down	up
M188T75_B_1	N,O-bisacetyl-serine	0.0025	1.045	0.0358	1.367	NA	NA	NA	up	down
M291T96_2_1	N-(Arginino)succinate	0.0022	1.277	0.0029	1.925	HMDB0000052	C03406	Carboxylic acids and derivatives	up	down
M174T50_1	N-Acetylaspartic acid	0.005	1.550	0.0187	1.936	HMDB0000812	C01042	Carboxylic acids and derivatives	up	down
M188T52_1	N-Acetylglutamic acid	7.41E-5	1.469	0.0308	1.402	HMDB0001138	C00624	Carboxylic acids and derivatives	up	down
M372T51_1	N-Acetylmuramic acid 6-phosphate	0.0075	1.607	0.0039	1.926	NA	C16698	NA	up	down

M148T81_A_1	N-Methyl-aspartic acid	1.18E-5	1.437	0.0105	1.509	HMDB0002393	C12269	Carboxylic acids and derivatives	up	down
M190T273_2_A_1	N-Phenylmaleamic acid	0.0014	1.399	0.029	1.402	NA	NA	NA	down	up
M399T392_1	N-Stearoyl Asparagine	6.00E-7	1.641	0.01	1.146	HMDB0241938	NA	Carboxylic acids and derivatives	up	down
M148T81_E_1	N-acetyl-serine	1.18E-5	1.437	0.0105	1.509	HMDB0002931	NA	Carboxylic acids and derivatives	up	down
M362T501_1	N-arachidonoylglycine	4.16E-4	1.355	0.0039	1.548	HMDB0005096	NA	Carboxylic acids and derivatives	up	down
M411T399_B_1	N-oleoyl-glutamine	2.32E-4	1.207	0.0161	1.089	HMDB0241958	NA	Carboxylic acids and derivatives	up	down
M581T49_A_1	Naringenin 8-C-alpha-rhamnopyranosyl-(1->2)-beta-glucopyranoside	1.98E-4	2.108	0.0202	2.085	HMDB0302090	NA	Flavonoids	down	up
M605T576_E_1	Neoannonin B	5.82E-5	2.252	0.0262	2.052	HMDB0037117	NA	Fatty Acyls	down	up
M195T394_B_1	Neolyratyl acetate	1.43E-4	1.024	0.0188	1.096	HMDB0041497	NA	Carboxylic acids and derivatives	down	up
M319T362_1	Noopept	5.39E-8	1.612	8.08E-4	1.356	HMDB0255714	NA	Carboxylic acids and derivatives	up	down
M581T49_B_1	Norrubrofusarin 6-beta-gentiobioside	1.98E-4	2.108	0.0202	2.085	HMDB0034570	NA	Naphthopyrans	down	up
M429T232_	Ononin	0.0022	1.260	0.0356	1.767	HMDB0033987	C10509	Isoflavonoids	up	down

1										
M281T316_A_1	Oxindanac	5.95E-4	1.502	0.0038	2.141	HMDB0255985	NA	Benzene and substituted derivatives	down	up
M285T308_A_1	Phenethyl caffeate	3.68E-4	1.001	0.0026	1.078	HMDB0246033	C10484	Cinnamic acids and derivatives	up	down
M428T398_1	Phospho-anandamide	1.08E-4	2.340	0.0145	1.863	NA	C19913	NA	up	down
M445T279_C_1	Physcion 8-glucoside	0.0048	1.331	0.0245	1.428	HMDB0303799	C10384	Anthracenes	down	up
M389T315_A_1	Piceid	2.12E-4	1.674	0.0017	2.005	HMDB0030564	C10275	Stilbenes	down	up
M159T255_A_1	Pimelic acid	1.81E-6	1.070	0.0057	1.103	HMDB0000857	C02656	Fatty Acyls	down	up
M425T288_A_1	Prostaglandin D2-1-glyceryl ester	5.79E-6	1.858	0.0407	1.054	HMDB0013653	NA	Fatty Acyls	up	down
M365T364_3_B_1	Prostaglandin E2 methyl ester	1.02E-6	1.842	0.0041	1.521	HMDB0256853	NA	Fatty Acyls	up	down
M349T329_A_1	Prostaglandin E3	9.29E-8	1.478	0.0013	1.296	HMDB0002664	C06439	Fatty Acyls	up	down
M425T288_C_1	Prostaglandin PGE2 1-glyceryl ester	5.79E-6	1.858	0.0407	1.054	HMDB0013043	NA	Fatty Acyls	up	down
M283T337_E_1	Prunetin	0.0031	1.550	0.016	1.993	HMDB0034127	C10521	Isoflavonoids	down	up
M130T64_1	Pyroglutamic acid	2.22E-5	1.387	0.0084	1.542	HMDB0000267	C01879	Carboxylic acids	up	down

								and derivatives		
M385T295_C_1	Recoflavone	4.23E-4	1.515	0.0099	1.719	HMDB0257131	NA	Flavonoids	down	up
M349T329_B_1	Resolvin E1	9.29E-8	1.478	0.0013	1.296	HMDB0010410	C18171	Fatty Acyls	up	down
M389T315_B_1	Resveratrol 4'-glucoside	2.12E-4	1.674	0.0017	2.005	HMDB0030565	NA	Stilbenes	down	up
M445T279_D_1	Rheochrysin	0.0048	1.331	0.0245	1.428	HMDB0035931	NA	Anthracenes	down	up
M605T576_C_1	Rollidecin D	5.82E-5	2.252	0.0262	2.052	HMDB0031886	NA	Fatty Acyls	down	up
M281T316_B_1	Rooperol	5.95E-4	1.502	0.0038	2.141	HMDB0257303	NA	Phenols	down	up
M394T516_1	S-geranylgeranyl-cysteine	2.97E-4	1.464	0.0282	1.353	NA	NA	NA	up	down
M795T472_B_1	Saponin E	0.0017	1.763	0.0061	2.329	HMDB0035958	NA	Prenol lipids	down	up
M223T288_1	Sinapate	0.0011	1.357	0.0276	1.335	HMDB0032616	C00482	Cinnamic acids and derivatives	down	up
M209T298_D_1	Sinapyl alcohol	8.90E-5	1.099	0.0219	1.057	HMDB0013070	C02325	Phenols	down	up
M942T461_B_1	Soyasaponin I	5.42E-4	1.677	0.0115	2.023	HMDB0034649	C08983	Prenol lipids	down	up
M912T463_B_1	Soyasaponin II	0.0016	1.694	0.036	1.875	HMDB0034650	C12081	Prenol lipids	down	up

M795T472_A_1	Soyasaponin III	0.0017	1.763	0.0061	2.329	HMDB0034651	C19865	Prenol lipids	down	up
M605T576_B_1	Squamocin L	5.82E-5	2.252	0.0262	2.052	HMDB0030940	NA	Fatty Acyls	down	up
M285T308_C_1	Stercurensin	3.68E-4	1.001	0.0026	1.078	HMDB0037485	NA	Linear 1,3-diarylpropanoids	up	down
M341T331_2_A_1	Tetramethoxyluteolin	0.002	1.177	0.0335	1.292	HMDB0301862	NA	Flavonoids	down	up
M283T337_D_1	Texasin	0.0031	1.550	0.016	1.993	NA	C10536	NA	down	up
M603T417_1	Tween 80	3.45E-6	1.875	0.0126	1.183	HMDB0256687	NA	Fatty Acyls	up	down
M309T223_2_1	Ubenimex	0.0074	1.015	0.0047	2.256	HMDB0242556	C00732	Peptidomimetics	up	down
M324T320_C_1	Undeca-2,4,6-trienoylcarnitine	0.0013	1.630	0.0299	1.796	HMDB0241198	NA	Fatty Acyls	down	up
M597T421_2_1	Urobilinogen	7.37E-6	2.025	0.0022	2.001	HMDB0004157	C05789	Tetrapyrroles and derivatives	up	down
M313T309_A_1	Yakuchinone A	4.34E-5	1.295	0.0074	1.245	HMDB0259946	C20211	Diarylheptanoids	up	down
M341T331_2_E_1	Zapotin	0.002	1.177	0.0335	1.292	HMDB0029461	NA	Flavonoids	down	up
M147T97_B_1	aldehydo-colitose	3.85E-5	1.175	4.46E-4	1.602	HMDB0248646	NA	Organooxygen compounds	down	up

M147T97_A_1	aldehydo-digitoxose	3.85E-5	1.175	4.46E-4	1.602	NA	NA	NA	down	up
M317T348_D_1	all-trans-18-Hydroxyretinoic acid	1.02E-7	1.474	0.0025	1.198	HMDB0012452	C16679	Prenol lipids	up	down
M317T348_C_1	all-trans-4-Hydroxyretinoic acid	1.02E-7	1.474	0.0025	1.198	HMDB0006254	C16677	Prenol lipids	up	down
M315T341_C_1	all-trans-4-Oxoretinoic acid	1.03E-7	1.473	0.0049	1.203	HMDB0006285	C16678	Prenol lipids	up	down
M317T348_E_1	all-trans-5,6-Epoxyretinoic acid	1.02E-7	1.474	0.0025	1.198	HMDB0012451	C16680	Prenol lipids	up	down
M273T404_A_1	alpha-Pyrufuran	1.13E-4	2.159	0.0199	1.153	HMDB0030635	C08929	Flavonoids	up	down
M273T404_B_1	beta-Pyrufuran	1.13E-4	2.159	0.0199	1.153	HMDB0030634	C08948	Benzofurans	up	down
M569T329_1	beta-galactosyl-cholate	7.06E-4	1.873	0.0128	1.123	NA	NA	NA	up	down
M385T295_A_1	beta-rhodomycinone	4.23E-4	1.515	0.0099	1.719	NA	NA	NA	down	up
M457T468_A_1	bryonolate	3.94E-4	1.421	0.0335	1.424	HMDB0036757	NA	Organooxygen compounds	down	up
M403T290_A_1	cis-Resveratrol 3-O-glucuronide	7.25E-5	1.459	0.0065	1.604	HMDB0041711	NA	Stilbenes	down	up
M403T290_B_1	cis-Resveratrol 4'-O-glucuronide	7.25E-5	1.459	0.0065	1.604	HMDB0041713	NA	Stilbenes	down	up
M605T576_	cis-Reticulatacin-10-one	5.82E-5	2.252	0.0262	2.052	HMDB0034967	NA	Fatty Acyls	down	up

D_1										
M385T295_E_1	dehydrodiconiferyl acid carboxylate	4.23E-4	1.515	0.0099	1.719	HMDB0041277	NA	2-arylbenzofuran flavonoids	down	up
M441T507_2_B_1	desaturated 7beta-hydroxythalianol	9.66E-4	1.199	0.0316	1.221	NA	NA	NA	down	up
M324T320_A_1	jasmonoyl-leucine	0.0013	1.630	0.0299	1.796	NA	NA	NA	down	up
M311T303_D_1	p-HBAD-I	3.28E-4	1.186	2.10E-4	2.189	NA	NA	NA	up	down
M225T236_C_1	phenyl ribotide	2.89E-7	1.325	0.0126	1.072	NA	NA	NA	down	up
M359T463_1	pregn-4-en-3-one-22-oate	1.14E-6	1.808	0.0029	1.454	NA	NA	NA	up	down
M375T299_1	proatranorin IV	0.0014	1.351	0.0144	1.543	NA	NA	NA	down	up
M425T288_B_1	prostaglandin E2 2-glyceryl ester	5.79E-6	1.858	0.0407	1.054	HMDB0013045	NA	Fatty Acyls	up	down
M351T317_1	prostaglandin I3	1.03E-7	1.278	0.0049	1.181	HMDB0256856	NA	Fatty Acyls	up	down
M405T299_E_1	salicyl salicylate glucoside	5.13E-4	1.327	0.0023	2.033	HMDB0031423	NA	Organooxygen compounds	down	up
M223T328_A_1	tetraketide 2-pyrone	8.31E-7	1.384	0.0023	1.397	NA	NA	NA	up	down
M148T81_B_1	threo-3-Methylaspartate	1.18E-5	1.437	0.0105	1.509	NA	C03618	NA	up	down

M445T279_E_1	tilianin	0.0048	1.331	0.0245	1.428	HMDB0259092	NA	Flavonoids	down	up
M445T279_A_1	wogonin 7-O-beta-glucoside	0.0048	1.331	0.0245	1.428	NA	NA	NA	down	up

Supplementary Table S3. Primer sequence list for qRT-PCR

**Gene**	**Forward primer (5'-3')**	**Reverse primer (5'-3')**
IL-1β	GGCTGGACTGTTTCTAATGC	ATGGTTTCTTGTGACCCTGA
TNF-α	GGGTGTTCATCCATTCTC	GGAAAGCCCATTTGAGT
NLRP3	ATTACCCGCCCGAGAAAGG	TCGCAGCAAAGATCCACACAG
Occludin	CCTGGAGGTACTGGTCT	ATCTTTCTTCGGGTTTT
Claudin-1	GGGGACAACATCGTGACCG	AGGAGTCGAAGACTTTGCACT
Cxcl1	CTGGGATTCACCTCAAGAACATC	CAGGGTCAAGGCAAGCCTC
GAPDH	GGTCTCCTCTGACTTCAACA	AGCCAAATTCGTTGTCATAC
